# Systems Immunology Characterization of Novel Vaccine Formulations for *Mycoplasma hyopneumoniae* Bacterins

**DOI:** 10.3389/fimmu.2019.01087

**Published:** 2019-05-24

**Authors:** Anneleen M. F. Matthijs, Gaël Auray, Virginie Jakob, Obdulio García-Nicolás, Roman O. Braun, Irene Keller, Rémy Bruggman, Bert Devriendt, Filip Boyen, Carlos A. Guzman, Annelies Michiels, Freddy Haesebrouck, Nicolas Collin, Christophe Barnier-Quer, Dominiek Maes, Artur Summerfield

**Affiliations:** ^1^Department of Reproduction, Obstetrics and Herd Health, Faculty of Veterinary Medicine, Ghent University, Merelbeke, Belgium; ^2^Institute of Virology and Immunology, Mittelhäusern, Switzerland; ^3^Department of Infectious Diseases and Pathobiology, Vetsuisse Faculty, University of Bern, Bern, Switzerland; ^4^Vaccine Formulation Laboratory, University of Lausanne, Epalinges, Switzerland; ^5^Interfaculty Bioinformatics Unit, Swiss Institute of Bioinformatics, University of Bern, Bern, Switzerland; ^6^Department of Biomedical Research, University of Bern, Bern, Switzerland; ^7^Laboratory of Veterinary Immunology, Department of Virology, Parasitology and Immunology, Faculty of Veterinary Medicine, Ghent University, Merelbeke, Belgium; ^8^Department of Pathology, Bacteriology and Avian Diseases, Faculty of Veterinary Medicine, Ghent University, Merelbeke, Belgium; ^9^Department of Vaccinology and Applied Microbiology, Helmholtz Centre for Infection Research, Brunswick, Germany

**Keywords:** *Mycoplasma hyopneumoniae*, bacterins, safety, immune responses, transcriptomics

## Abstract

We characterized five different vaccine candidates and a commercial vaccine in terms of safety, immunogenicity and using a systems vaccinology approach, with the aim to select novel vaccine candidates against *Mycoplasma hyopneumoniae*. Seven groups of six *M. hyopneumoniae*-free piglets were primo- and booster vaccinated with the different experimental bacterin formulations, the commercial vaccine Hyogen® as a positive control or PBS as a negative control. The experimental bacterin was formulated with cationic liposomes + c-di-AMP (Lipo_AMP), cationic liposomes + Toll-like receptor (TLR) 2/1, TLR7, and TLR9 ligands (TLR ligands; Lipo_TLR), micro-particles + TLR ligands (PLGA_TLR), squalene-in-water emulsion + TLR ligands (SWE_TLR), or DDA:TDB liposomes (Lipo_DDA:TDB). Lipo_DDA:TDB and Lipo_AMP were the most potent in terms of serum antibody induction, and Lipo_DDA:TDB, Lipo_AMP, and SWE_TLR significantly induced Th1 cytokine-secreting T-cells. Only PLGA_TLR appeared to induce Th17 cells, but was unable to induce serum antibodies. The transcriptomic analyses demonstrated that the induction of inflammatory and myeloid cell blood transcriptional modules (BTM) in the first 24 h after vaccination correlated well with serum antibodies, while negative correlations with the same modules were found 7 days post-vaccination. Furthermore, many cell cycle and T-cell BTM upregulated at day seven correlated positively with adaptive immune responses. When comparing the delivery of the identical TLR ligands with the three formulations, we found SWE_TLR to be more potent in the induction of an early innate immune response, while the liposomal formulation more strongly promoted late cell cycle and T-cell BTM. For the PLGA formulation we found signs of a delayed and weak perturbation of these BTM. Lipo_AMP was found to be the most potent vaccine at inducing a BTM profile similar to that correlating with adaptive immune response in this and other studies. Taken together, we identified four promising vaccine candidates able to induce *M. hyopneumoniae*-specific antibody and T-cell responses. In addition, we have adapted a systems vaccinology approach developed for human to pigs and demonstrated its capacity in identifying early immune signatures in the blood relating to adaptive immune responses. This approach represents an important step in a more rational design of efficacious vaccines for pigs.

## Introduction

*Mycoplasma hyopneumoniae (M. hyopneumoniae)* is the primary cause of enzootic pneumonia (EP), a chronic respiratory disease in pigs. The disease causes severe economic losses in swine-producing countries worldwide due to a reduced average daily weight gain of the pigs, a higher feed conversion ratio and an increased use of antimicrobial agents (1–3). Control of the disease can be achieved by optimizing management and housing conditions combined with medication and vaccination (2).

Vaccination with inactivated, adjuvanted whole-cell bacterins is practiced worldwide to control EP (4). However, current commercial vaccines only offer partial protection, have a limited effect on the transmission of the microorganism and cannot prevent colonization (5–7). Most commercial bacterins are based on the J-strain, a low virulent *M. hyopneumoniae* strain isolated in the UK in the sixties (8–10), and contain adjuvants including aluminum hydroxide, carbopol, mineral oil or biodegradable oil (4). The main effects of vaccination are a reduction in clinical symptoms, lung lesions, medication use, and performance losses (11, 12). Those effects may vary between pig herds (2), which could be partially explained by antigenic and pathogenic differences between the strains circulating in the herd and the vaccine strain (10).

The immune mechanisms leading to protection against *M. hyopneumoniae* infection are complex and not yet fully elucidated. *M. hyopneumoniae*-specific serum antibody concentrations induced by vaccination are not correlated with the severity of lung lesions in *M. hyopneumoniae*-infected pigs (5, 13), indicating that systemic antibodies play only a minor role in protective immunity. However, local mucosal antibodies (IgA) are considered important to prevent and control *M. hyopneumoniae*-induced pneumonia, as the adherence of the microorganism to the ciliated epithelium of the respiratory tract is the first step in the pathogenesis (14). Also, several studies suggest that systemic cell-mediated immune responses play a major role in disease protection (14–17).

Based on this knowledge, innovative bacterin formulations that include virulent *M. hyopneumoniae* strains formulated with adjuvants specifically designed to promote cellular immune responses could improve vaccine efficacy. Therefore, we developed three different vaccine formulations to deliver a cocktail of TLR 2/1, TLR 7, and TLR 9 ligands previously shown to potently activate porcine antigen presenting cells including dendritic cells (DC), monocytes and B cells (18, 19). The formulations included a liposomal, a micro-particle and an oil-in-water formulation. In addition, we developed a liposomal formulation to deliver a cyclic di-nucleotide targeting the STING pathway (20) as an alternative immunostimulant, and another cationic liposomal formulation to deliver a Mincle ligand, also previously found to be efficacious (21). All formulations were based on the *M. hyopneumoniae* strain F7.2C, a highly virulent field strain isolated in Belgium in 2000 (22, 23), and shown to be antigenically different from the J-strain (23).

Overall, the aim of this study was to assess the safety of these five novel bacterin formulations and characterize the immune responses induced by the formulations, compared to a commercial vaccine in order to select new promising vaccine candidates. To this end, *M. hyopneumoniae-*specific T cell responses and antibody responses were measured in pigs. For T cells, we focussed on Th1 and Th17 based on their known role in protective immunity against *Mycoplasma* infection, as identified in mouse models (24). Next to that, we employed a systems immunology approach to understand how different formulations modulate the immune system toward potent immunogenicity. This analysis employed “blood transcriptional modules” (BTM) defined for peripheral blood cells in human (25), which were adapted to pigs. This technique sheds light into the black box of the immune response by identifying pathways and networks of genes related to adaptive immune responses as previously demonstrated for human and sheep (25–34). Also, this approach has been shown to possess more discriminative power for analyses of peripheral blood leukocytes during vaccination when compared to gene sets based on canonical pathways (25). Our work has demonstrated the possibilities of such novel approaches in vaccinology and identified vaccine candidates for further exploration.

## Materials and Methods

### Vaccines

The vaccine strain *M. hyopneumoniae* F7.2C was grown in modified Friis medium (35) for 5 days at 37°C. The culture, containing 5 × 10^8^ color changing units (CCU)/ml, was inactivated by incubation with 4 mM binary ethyleneimine (BEI) under agitation at 37°C for 24 h. Subsequently, the BEI was neutralized by incubating the inactivated culture with 4 mM sodium thiosulfate under agitation at 37°C for 24 h. Inactivated bacteria were pelleted at 15,000 g for 40 min at 4°C and washed three times in 50 ml sterile phosphate buffered saline (PBS). The final pellet was resuspended in sterile PBS.

For this study, five adjuvant formulations were developed based on the association of particle-based delivery systems [liposomes, poly(lactic-co-glycolic acid) [PLGA] microparticles and a squalene-in-water emulsion (SWE)] with different immune stimulators. These included the Mincle agonist trehalose 6,6-dibehenate (TDB, Avanti, Alabaster, AL, USA), the STING ligand cyclic diadenylate monophosphate (c-di-AMP, produced at the Helmholtz Center for Infection Research, Braunschweig, Germany) and a combination of TLR ligands: TLR1/2 ligand Pam3Cys-SK4 (PAM, EMC Microcollections, Tübingen, Germany), TLR9 ligand CpG ODN SL03 (CpG, Eurofins Genomics, Les Ulis, France), and TLR7/8 ligand resiquimod (Chemdea, Ridgewood, NJ, USA).

Two cationic liposome formulations were produced, based on the thin lipid film method (36), and followed by extrusion: TDB was combined with dimethyl dioctadecylammonium (DDA) bromide to form Lipo_DDA:TDB, and c-di-AMP was encapsulated into 1,2-dipalmitoyl-sn-glycero-3-phosphocholine and dimethylaminoethane-carbamoyl-cholesterol (DPPC:DC-Chol) cationic liposomes (37) to obtain Lipo_AMP. The TLR ligand selection was combined in different delivery systems: PLGA micro-particles, cationic liposomes and SWE. Cationic liposomes (DPPC:DC-Chol) and PLGA cationic micro-particles (combined to ethylaminoethyl-dextran) were produced with the thin lipid film and the double emulsion (W/O/W) methods (38), respectively. Pam3Cys-SK4 and resiquimod were encapsulated into both types of particles and CpG was later adsorbed to their surface to form the Lipo_TLR and PLGA_TLR formulations. Finally, for the SWE_TLR formulation, SWE [a squalene-based formulation developed and produced by the Vaccine Formulation Laboratory, and composed of 3.9% (w/v) squalene, 0.5% (w/v) Tween 80 and 0.5% (w/v) Span 85 (39)] was mixed with the same immune stimulators (PAM, CpG, and resiquimod).

For each formulation we measured the following physico-chemical characteristics: particle size (PS), polydispersity index (Pdi), and zeta potential (ZP), by means of dynamic light scattering for the liposomes and SWE, and laser diffraction for the micro-particles (Zetasizer Nano ZS and Mastersizer 3000, Malvern, UK). The amounts of immune-stimulators loaded into the Lipo_AMP and Lipo_TLR formulations were indirectly determined by the use of nanodrop (for c-di-AMP) and fluorescently labeled immune-stimulator (CpG-FITC and Pam-Rodhamine, Invivogen, San Diego, CA, USA) methods. The free immune-stimulators were separated from the liposomes by filtration using the Vivaspin® 500 centrifugal concentrator (PED membrane, MWCO 100 kDa, Sartorius, Göttingen, Germany) and then quantified as mentioned above (Supplementary Table 1) Antigen was mixed with the final product, and PS and ZP of the formulations were monitored over a period of 1 month.

The composition of each experimental vaccine is given in Table 1. The commercial vaccine employed was Hyogen® (CEVA Santé Animale, Libourne Cedex, France) representing a mineral oil emulsion with *Escherichia coli* J5 non-toxic LPS as immunostimulant and inactivated *M. hyopneumoniae* field isolate BA 2940-99 as antigen.

**Table 1:** Composition of the experimental *M. hyopneumoniae* bacterins and their route of administration.

**Vaccine formulation**	**Dose (mL)**	**Delivery system**	**Immune-stimulators (μg/dose)**	**Antigen dose (CCU)**	**Administration route**	
					**Primo**	**Booster**
Lipo_AMP	2	DPPC:DC-Chol liposomes	C-di.AMP (100)	10^9^	IM	IM
Lipo_TLR	2	DPPC:DC-Chol liposomes	Pam3Cys-SK4/CpG ODN SL03/resiquimod (80/80/80)	10^9^	IM	IM
PLGA_TLR	2	PLGA micro-particles (combined to ethylaminoethyl-dextran)	Pam3Cys-SK4/CpG ODN SL03/resiquimod (80/80/80)	10^9^	IM	IM
SWE_TLR	2	squalene-in-water emulsion	Pam3Cys-SK4/CpG ODN SL03/resiquimod (80/80/80)	10^9^	IM	IM
Lipo_DDA:TDB	IM 2 ID 0.2	DDA liposomes	TDB (500)	IM 10^9^ ID 2x10^8^	IM+ID	IM

*CCU, color changing units; IM, intramuscular; ID, intradermal; DPPC:DC-Chol, 1,2-dipalmitoyl-sn-glycero-3-phosphocholine and dimethylaminoethane-carbamoyl-cholesterol; c-di-AMP, bis-(3′,5′)-cyclic dimeric adenosine monophosphate; PLGA, poly(lactic-co-glycolic acid); DDA, dimethyl dioctadecylammonium; TDB, trehalose 6,6′-dibehenate*.

### Animal Experiment

The study was performed after approval by the Ethical Committee for Animal Experiments of the Faculty of Veterinary Medicine, Ghent University (approval number EC2016/91). Forty-two *M. hyopneumoniae*-free Rattlerlow-Seghers piglets (RA-SE Genetics NV, Ooigem, Belgium) were enrolled in the study. All animals were purchased from a herd that has been free of *M. hyopneumoniae* for many years based on repeated serological testing, nested PCR testing on tracheobronchial swabs, and absence of clinical signs and pneumonia lesions in the slaughter house. The piglets were weaned at 28 days of age and transported 4 days later to the experimental facilities of the Faculty of Veterinary Medicine, Ghent University, Belgium. They were housed in stables with absolute air filters for impending particles (HEPA U15) on both incoming and outgoing ventilation shafts and fed *ad libitum* with a non-antimicrobial-supplemented diet. On the day of arrival at the experimental facilities, the piglets were randomly allocated into six vaccination groups and one control group of six piglets each. Due to practical reasons, the piglets were vaccinated, sampled and euthanized over 2 consecutive days. After an acclimatization period of 6 days, the piglets of the vaccination groups were primo-vaccinated (D0; 39–40 days of age) intramuscularly (IM) into the right side of the neck with 2 mL vaccine. Additionally, group Lipo_DDA:TDB was vaccinated intradermally (ID) into the left side of the neck with 0.2 mL vaccine. The rationale for the ID injection of formulation Lipo_DDA:TDB was based on a previous report showing that CAF01, a liposome-based adjuvant containing similar immunomodulators, was able to induce mucosal immunity when administered this way (40). The piglets of the control group were injected IM into the right side of the neck with 2 mL sterile PBS. Two weeks later (D14), the piglets of the vaccination groups were booster vaccinated IM with 2 mL vaccine (all groups). The control group received 2 mL PBS IM. On D28 all piglets were euthanized.

### Safety Parameters

The piglets were observed daily for at least 15 min from D-6 until D28 of the study. On the days of vaccine administration, the piglets were observed twice: shortly before (D0; D14) and 4 h after vaccination (D0+4h; D14+4h). For each piglet, clinical findings regarding body condition (skinny), behavior (depressed, unconscious), respiration (sneezing, coughing, abdominal breathing), digestion (diarrhea, vomiting), lameness and other remarkable findings were recorded. At necropsy (D28), lungs were macroscopically examined for the presence of lesions according to Hannan et al. (41). Subsequently, bronchoalveolar lavage (BAL) fluid was collected from one lung part by flushing the head bronchus with 20 mL sterile PBS, as previously described (15). From the BAL fluid, DNA was extracted using a commercial kit (DNeasy^®^ Blood & Tissue kit, Qiagen, Venlo, The Netherlands) and a nested PCR for the detection of *M. hyopneumoniae* DNA was performed according to Stärk et al. (42).

The pigs were weighed on the day of primo-vaccination (D0) and at euthanasia (D28). Average daily gain (ADG) in g/pig/day was calculated according to Sacristán et al. (43).

Rectal body temperature was measured shortly before and 4 h after vaccine administration, then daily until 4 days post-vaccine administration, and on D7, 10, 21, 24, and 28 of the study. This was based on the guidelines on safety evaluation of veterinary vaccines written in the European Pharmacopeia 8.0.

Injection site reactions (ISR) were evaluated shortly before vaccination, 4 h after vaccination and then daily from D1 to D28 using the scoring system explained in Supplementary Table 2. Scores could range from 0 to 3 with 0 = normal, 1 = mild, 2 = moderate, and 3 = severe. At euthanasia (D28), tissue samples from the injection site were collected from all study animals for histopathological examination. All IM and ID injection sites were marked with a permanent pen upon vaccination. Out of the marked area a tissue sample of approximately 2 cm^2^ with a depth of 5 cm (IM injection site) or 3 cm (ID injection site) was removed in an angle of 90° to the skin. A tissue sample with a dimension of 2 × 2 × 3 cm from the left side of the neck was collected as described above from the pigs of the control group to serve as a control for the ID injection sites. The tissues were fixed immediately after sampling in 10% neutral formalin. After fixation, tissue blocks were sectioned from the samples, embedded in paraffin and histological slides were stained with hematoxylin and eosin. Each injection site sample was evaluated using light microscopy and an overall score ranging from 0 to 3 (0 = not detected, 1 = mild, 2 = moderate, and 3 = severe) was given. This score took into account the presence and degree of hemorrhage, blood resorption, necrosis, inflammation (acute and chronic), angiogenesis, and proliferation of connective tissue.

### Serology

Before primo-vaccination (D0), on D7, on the day of booster vaccination (D14) and at euthanasia (D28), serum samples were collected and analyzed for the presence of antibodies against *M. hyopneumoniae* with a commercial blocking ELISA (IDEIA™ *Mycoplasma hyopneumoniae* EIA kit, Oxoid Limited, Hampshire, UK) according to the manufacturer's instructions. Samples with optical density (OD) lower than 50% of the average OD of the buffer control were considered positive. Samples with OD-values equal or bigger than 50% of the average OD of the buffer control were classified as negative.

Immunoglobulin (Ig) G and IgA isotypes of the *M. hyopneumoniae*-specific antibodies in serum were determined with an in-house indirect ELISA. Briefly, Nunc Maxisorb® flat-bottom 96 well plates (eBioscience, San Diego, CA, USA) were coated overnight at room temperature with Tween 20-extracted *M. hyopneumoniae* antigens (44). After blocking with PBS containing 0.05% Tween 20 and 1% BSA for 2 h at 37°C, plates were washed three times with PBS + 0.05% Tween 20 and serum diluted 1:200 and 1:100 was added for the detection of IgG and IgA, respectively. After incubating for 30 min at 37°C, plates were washed again, and peroxidase-labeled goat anti-porcine polyclonal IgG diluted 1:60,000 and IgA diluted 1:20,000 (Bethyl Laboratories, Montgomery, TX, USA) were added. Plates were incubated again for 30 min at 37°C, washed and 3,3′5,5′-tetramethylbenzidin substrate (Sigma-Aldrich, Saint Louis, MO, USA) was added. After incubating for 10 min, the reaction was stopped with 2 N HCl and the OD was measured at 450 nm. All samples were tested in duplicate. To relatively quantify the antibody levels a standard curve was made using two-fold serial dilutions of a positive reference serum corresponding to defined arbitrary units (1:800 dilution defined as 1 unit). The interpolation from the standard curve employed non-linear regression with least square fits using Graphpad Prism 7.0 (GraphPad Software Inc., San Diego, CA, USA).

### *M. hyopneumoniae*-Specific Antibodies in Bronchoalveolar Lavage (BAL) Fluid

The BAL fluid collected on D28 was analyzed undiluted for the presence of *M. hyopneumoniae*-specific IgA antibodies using peroxidase-labeled goat anti-porcine polyclonal IgA (Bethyl Laboratories, Montgomery, TX, USA) diluted 1:80,000 in an in-house indirect ELISA as described above. A cut-off was calculated as mean OD-value from the control animals plus three times the SD and established at an OD-value of 0.098. Samples with OD-values higher than the cut-off were considered positive and samples equal to or below the cut-off were considered negative.

### T Cell Assays

Shortly before the booster vaccination on D14 and on the day of euthanasia (D28), blood samples were taken from each animal to assess the primary and secondary T cell-specific responses against *M. hyopneumoniae*. For each animal, samples were restimulated in triplicate cultures and analyzed separately. Briefly, peripheral blood mononuclear cells (PBMCs) were isolated using a ficoll-plaque density gradient (1.077 g/L, GE Healthcare Bio-sciences Corp., Piscataway, NJ, USA) and plated in 12-well plates at 5 × 10^6^ cells/well in 1 ml of AIM-V medium (Gibco™, ThermoFisher Scientific, Waltham, MA, USA). Subsequently, the cells were restimulated *in vitro* overnight (18 h) with 6.25 × 10^7^ CCU/mL of *M. hyopneumoniae* F7.2C bacterin. For the last 4 h of stimulation, we added Brefeldin A (eBioscience, San Diego, CA, USA) in each well to inhibit cytokine release and allow intra-cellular detection of cytokines by flow cytometry (FCM). Concanavalin A stimulation (10 μg/mL, Sigma-Aldrich, Saint Louis, MO, USA) was employed as a positive control. Cells were then harvested and the cytokine production of T cell populations was determined by FCM, using a 5-step 6-color staining protocol. Cells were first incubated with the LIVE/DEAD™ Fixable Aqua Dead Cell Stain Kit (Invitrogen™, ThermoFisher Scientific, Waltham, MA, USA) according to the manufacturer's instructions. The cells were then incubated with anti-CD4 (clone 74-12-4, Southern Biotech, Birmingham, AL, USA) and anti-CD8β (clone PG164A, WSU, Pullman, WA, USA) antibodies, and subsequently with the corresponding secondary antibodies: anti-mouse IgG2b AlexaFluor 488 (Molecular Probes, Eugene, OR, USA) and anti-mouse IgG2a PE-Cy7 (Abcam, Cambridge, UK), respectively. Following surface staining, cells were fixed and permeabilized using the BD Cytofix/Cytoperm™ Fixation/Permeabilization Solution kit (Becton Dickinson, Franklin Lakes, NJ, USA) according to the manufacturer's instructions. Cells were finally incubated with directly coupled anti-human TNF-α AlexaFluor 647 (clone MAb11, BioLegend, San Diego, CA, USA), anti-pig IFN-γ PerCP-Cy5.5 (clone P2G10, Becton Dickinson, Franklin Lakes, NJ, USA), and anti-human IL-17A PE (clone SCPL1362, Becton Dickinson, Franklin Lakes, NJ, USA). Flow cytometry acquisition was performed on a CytoFLEX flow cytometer (Beckman Coulter, Brea, CA, USA) and the results were further analyzed with the FlowJo™ software (Tree Star Inc., Ashland, OR, USA).

### Vaccine-Induced Transcriptional Responses

Blood samples were collected on D0, D1, and D7 for RNA preparation (2.5 ml in PAXgene® Blood RNA Tubes, Becton Dickinson, Franklin Lakes, NJ, USA). RNA was extracted using the Paxgene® Blood RNA kit (Qiagen, Venlo, The Netherlands) and the RNA quality was controlled with a Fragment Analyzer. All samples were found to have good quality [RNA integrity number (RIN) > 8] and were sequenced using an Illumina® HiSeq 3000 sequencer (Illumina, San Diego, CA, USA). The quality of the reads was assessed using FastQC v. 0.11.2^1^ The reads were mapped to the *Sus scrofa* reference genome (Sscrofa_11.1) with HISAT2 v. 2.1.0 (45). Feature Counts from Subread v. 1.5.3 was employed to count the number of reads overlapping with each gene, as specified in the Ensembl annotation build 91. The RNAseq data are available in the European Nucleotide Archive^2^ under the accession number PRJEB30361.

The Bioconductor package DESeq2 v. 1.18.1 was used to test for differential gene expression between the different time points for each vaccine separately (46). Our specific interest was to identify genes where the change between two time points was different in vaccinated animals compared to the controls. Therefore, a two-factorial model was used, including the factors time point and group (vaccine vs. control), and their interaction. The genes were then ranked based on the P-values for the interaction term for a “ranked gene set enrichment analysis” (GSEA) (47) using the BTM as defined by Li et al. (48).

The BTM were adapted to the pig by replacing human genes with their pig homologs. This step involved extensive manual curation. The final lists of genes for each module can be found in the Data Sheet 1.

To compare the module activity of the different vaccines, all modules with a false discovery rate (FDR) *q* < 0.1 were used. In GSEA, a cut-off of 0.25 is recommended but in this study a cut-off of 0.1 was selected to reduce the amount of BTM changing over time. Heat maps were created reflecting the modular activity calculated as the negative natural logarithm of the *P*-value. For negative enriched BTM, this was multiplied with −1 to obtain a positive value. The rationale of this was to obtain a value reflecting both the enrichment of a module and its statistical significance.

### Correlation Analyses of BTM and Vaccine-Induced Adaptive Immune Responses

To get more insight in the immunomodulation toward a potent immune response, BTM were correlated with the vaccine-induced adaptive immune responses (antibodies, *M. hyopneumoniae*-specific INFγ^+^TNF^+^ CD4 T cells and CD8 T cells). To this end, single-sample (ss) GSEA scores were first calculated to transform a single sample's gene expression profile to a gene set (BTM) enrichment profile^3^ as described in Barbie et al. (49). Subsequently, the time-dependent changes in ssGSEA values for each BTM were determined as the ratio of D1:D0, D7:D0, and D1:D7 ssGSEA values. These ratios were then correlated to the immune response values using Pearson's correlation coefficient. In order to obtain sufficient values, the data from all vaccinated animals (controls excluded) was used. Only correlation coefficients with *P* < 0.05 were considered.

### Statistical Analyses

Fisher's exact tests were performed to analyse differences in the number of animals with ISR and histopathological findings (irrespective of type) at the injection site between the control group and the vaccinated groups. A Bonferroni correction for multiple tests was applied. Rectal temperature values were averaged for the following periods: D1-3, D4-14, D15-17, and D18-28 to distinguish between systemic reactions shortly after vaccination (D1-3; D15-17) and systemic reactions developed later on (D4-14; D18-28). Rectal temperature and ADG were not normally distributed according to the Shapiro-Wilk's test, and Mann-Whitney U tests were run to analyse differences between the control and vaccinated groups in ADG, rectal temperature measured 4 h after vaccination (D0+4h; D14+4h) and during the following periods: D1-3, D4-14, D15-17, D18-28. The Bonferroni method was applied to correct for multiple comparisons. For the quantitative antibody ELISA and T cell data, a two-way ANOVA was employed using the factors vaccine and time. Tukey's or Dunnett's tests were used to correct for multiple comparisons, respectively. Statistical analyses of clinical variables were conducted in SPSS 24 for Windows (IBM, Armonk, NY, USA) and for immune response data using GraphPad Prism 7.0 (GraphPad Software Inc., San Diego, CA, USA). Significance is indicated as ^*^P ≤ 0.05; ^**^P < 0.01; ^***^P < 0.001.

## Results

### Safety of the Vaccines

To evaluate the safety of the vaccines the general health, ADG and rectal temperature of the piglets was closely monitored. Diarrhea, which sometimes resulted in skinny pigs, was the most frequent clinical finding observed in all groups (Lipo_DDA:TDB: 2/6; Hyogen and PLGA_TLR: 4/6; control, Lipo_TLR, SWE_TLR: 5/6; Lipo_AMP: 6/6; Supplementary Figure 1B). As it was mostly seen during the acclimatization period and started the day after arrival, it was diagnosed as post-weaning diarrhea. All pigs were treated once with 5 mg enrofloxacin per kg body weight (Floxadil® 50 mg/mL, Emdoka, Hoogstraten, Belgium) IM in the hind leg and responded well on treatment. Arthritis (swollen joints) was also observed (control, Lipo_AMP, Lipo_TLR, PLGA_TLR: 1/6; Hyogen: 3/6) and cases occurred during the whole study period (Supplementary Figure 1C). Bursitis was recorded for one pig in groups Lipo_TLR, SWE_TLR and Hyogen, and lameness for one pig in groups Lipo_AMP and Lipo_DDA:TDB (Supplementary Figures 1D–E). Behavior and respiration were normal throughout the entire study, except for one pig of the PLGA_TLR group that showed severe abdominal breathing following blood sampling on D14. At necropsy (D28), none of the pigs had macroscopic lung lesions and no *M. hyopneumoniae* DNA was detected in BAL fluid. The vaccinated groups did not differ in ADG compared to the control group (data not shown). Four hours after primo- and booster vaccination (D0+4h, D14+4h), rectal temperatures of groups SWE_TLR and Hyogen were significantly higher compared to the control group (*P* ≤ 0.05). Rectal temperatures from Lipo_AMP and Lipo_TLR were also increased over the physiological threshold (>40°C) 4 h after primo- and booster vaccination. However, this increase was only statistically significant compared to the control group at D0+4h and D14+4 for groups Lipo_TLR and Lipo_AMP, respectively (*P* ≤ 0.05). A slight increase, although not statistically significant, was observed for PLGA_TLR 4 h after primo-vaccination. Group means were back to normal 1day after vaccination and all remained within normal physiological levels during the remainder of the trial (Supplementary Figure 1F).

The presence and severity of ISR was recorded daily and a histopathological examination of each injection site was performed at the end of the study (D28). No ISR were seen in the control group and group PLGA_TLR. Overall mild and transient ISR were observed in one pig of group SWE_TLR, and in two pigs of each of the groups Lipo_AMP, Lipo_TLR, and Hyogen. In the group Lipo_DDA:TDB three pigs showed a moderate but transient ISR at the IM injection site. However, at the ID injection site, all pigs showed a prolonged mild to moderate ISR which lasted until the end of the study in 4/6 pigs. A more detailed overview of the duration of the ISR and their severity is given in Supplementary Table 3. Histopathological examination of the injection site at D28 revealed an overall severe foreign body reaction with chronic inflammation, angiogenesis, and proliferation of connective tissue in 5/6 ID injection site samples from group Lipo_DDA:TDB. Mild (moderate for one pig in group SWE_TLR) focal chronic inflammation was observed in all IM injected groups. Mild to moderate hemorrhage was also observed in all IM injected groups. This was probably caused by the sampling itself as it was most of the time located at the borders of the collected tissue. The results of the histopathological examination of the injection sites are represented in Supplementary Table 4.

### *M. hyopneumoniae*-Specific Antibody Responses

According to the commercial blocking ELISA (Oxoid; Supplementary Table 5), all pigs from the control group remained serologically negative for *M. hyopneumoniae* throughout the study. On day 28 of the study, all the animals from groups Lipo_AMP, Lipo_TLR, SWE_TLR, Lipo_DDA:TDB, and Hyogen were seropositive. From the PLGA_TLR group, only two out of six pigs seroconverted at D28.

**Figure 1:**
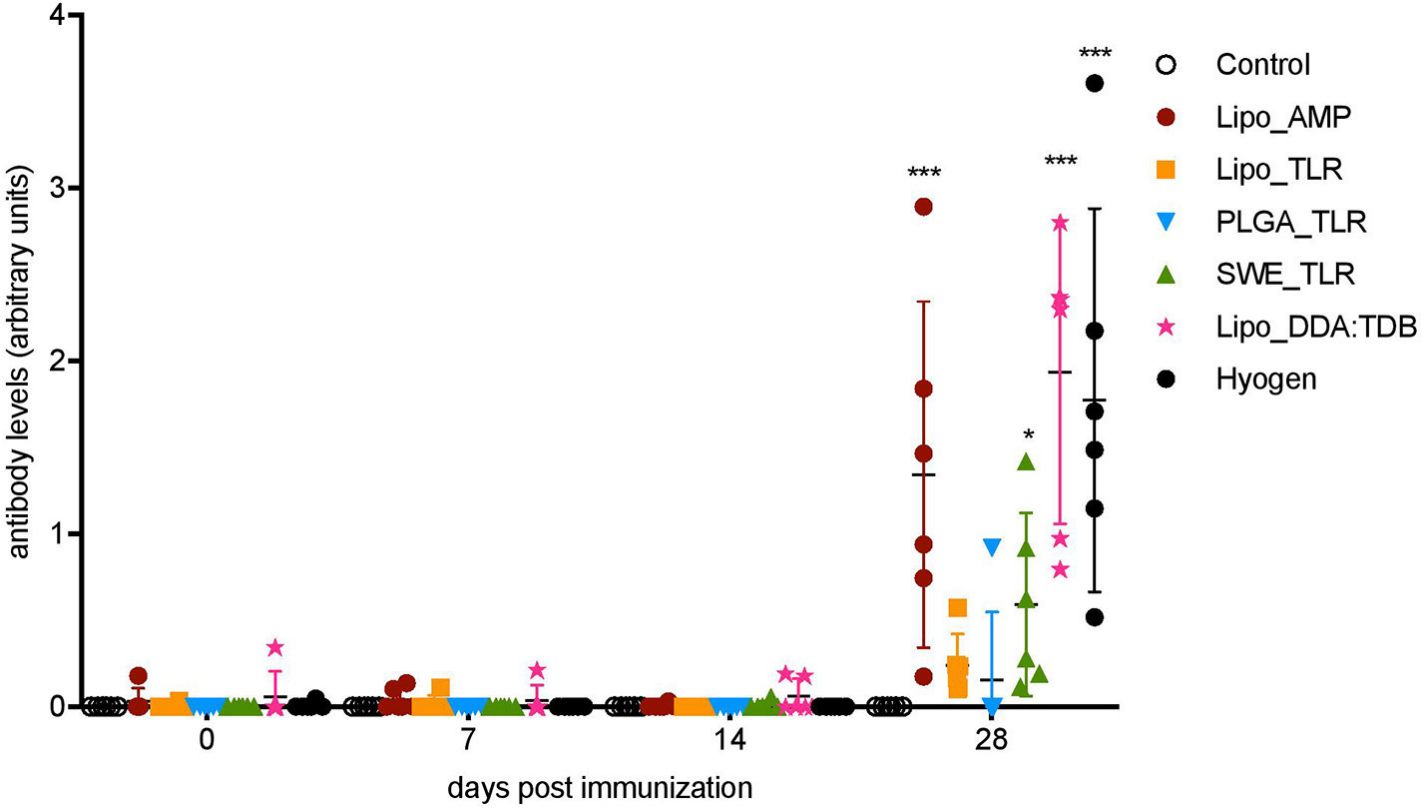
Serum antibody levels following vaccination of pigs with vaccine candidates. *M. hyopneumoniae-*specific IgG antibodies induced by the five novel vaccines and one commercial vaccine listed in the legend were determined by indirect ELISA. Six animals per group received two injections in 14 days interval. Individual animals are shown. Significance was calculated using two-way ANOVA followed by Tukey's test (^*^P < 0.05; ,^**^P < 0.01; ^***^P < 0.001).

To quantify serum IgG levels in arbitrary units we used an in-house indirect ELISA with a positive reference serum as a standard (Figure 1). At D28, groups Lipo_AMP, SWE_TLR, Lipo_DDA:TDB, and Hyogen were statistically different from the control group. The Lipo_DDA:TDB formulation induced the highest IgG response, followed by the Hyogen and Lipo_AMP formulations. Group Lipo_TLR was not significantly higher than the control group, although we could detect *M. hyopneumoniae*-specific IgG antibodies in all animals from this group. In the PLGA_TLR group, only one animal appeared to react. No *M. hyopneumoniae*-specific IgA antibodies were observed for any of the groups at any time point in the serum. Only one animal from the SWE_TLR group was positive for *M. hyopneumoniae*-specific IgA in BAL fluid on D28 (Supplementary Table 3).

### T Cell Responses

**Figure 2:**
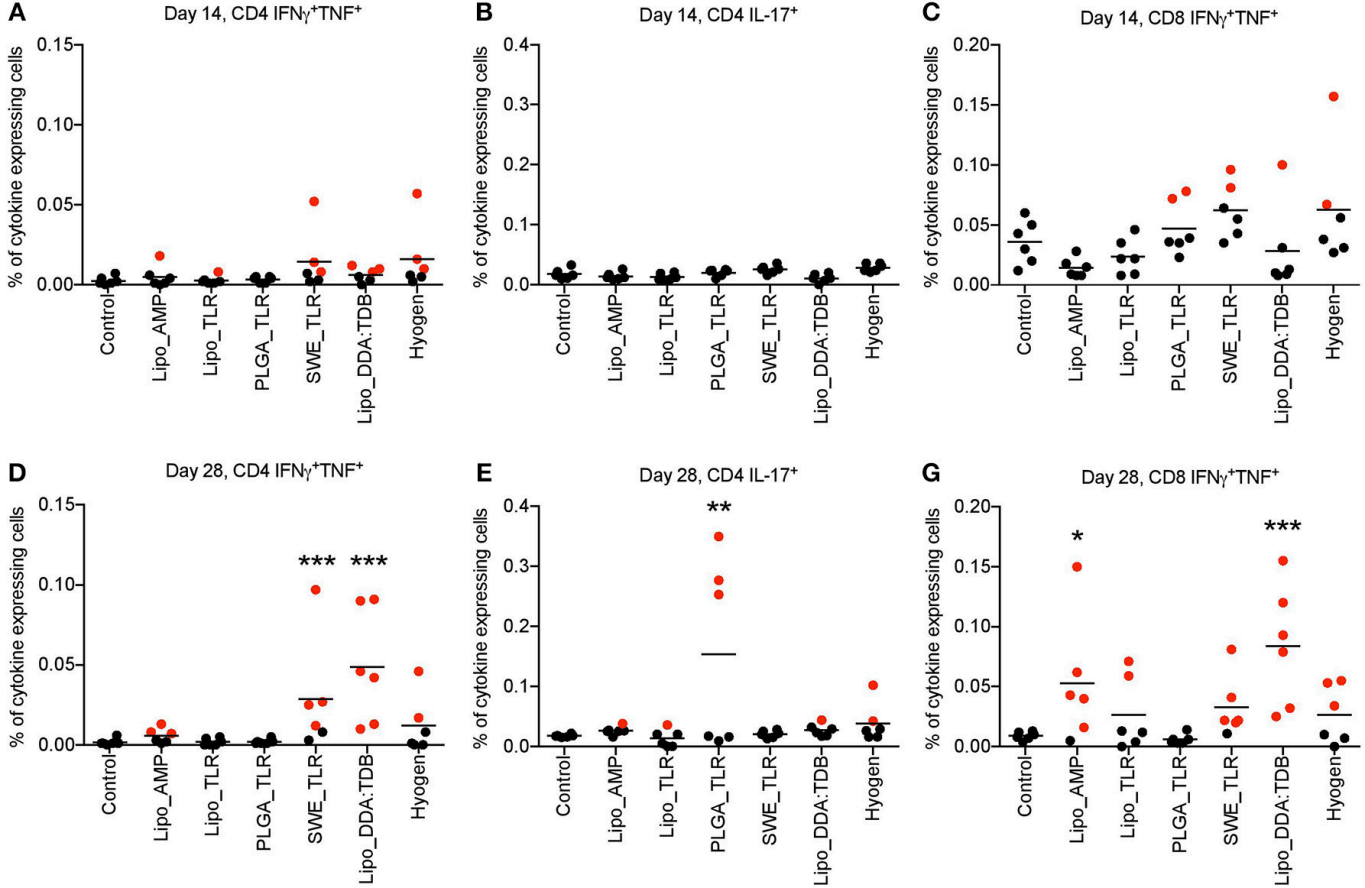
*M. hyopneumoniae-*specific T cell responses induced following vaccination of pigs with vaccine candidates. Six animals per group were prime-boost vaccinated on D0 and D14. At D14 and D28, *M. hyopneumoniae*-specific T cells induced by the tested vaccines listed in the legend were determined by *in vitro* restimulation of PBMC from vaccinated animals followed by intracellular cytokine staining and multicolor flow cytometry. Following doublet exclusion, live cells were gated and the percentage of IFNγ^+^TNF^+^ double positive CD4^+^ **(A,D)** and CD8β^+^ **(C,F)** T cells as well as IL-17A^+^ CD4 T cells **(B,E)** was determined. The mean values obtained from triplicate cultures for individual animals are shown. Positive animals are marked in red (defined as being above the 99% CI of the control group). Significance was calculated using two-way ANOVA followed by Dunnett‘s test (^*^P < 0.05; ^**^P < 0.01; ^***^P < 0.001). PBMC, peripheral blood mononuclear cells.

The results of the *M. hyopneumoniae*-specific T cell responses after primo-vaccination (D14) are presented in Figures 2A–C. No significant group differences were found for the percentage of cytokine-producing T cells in the peripheral blood compartment. Nevertheless, as antigen-specific T cells are transient in the blood, a negative result cannot be interpreted as a lack of T cell response. In fact, a few animals appeared to respond (defined as being above the 99% confidence interval (CI) of the control group) indicating some degree of T cell priming in certain groups. This was found in particular in the groups SWE_TLR, Lipo_DDA:TDB and Hyogen with three animals above this threshold for the TNF^+^IFN-γ^+^ double positive CD4 (Th1) cells (Figure 2A). For the CD8^+^ TNF^+^IFN-γ^+^ T cells, two animals were above the threshold in the PLGA_TLR, SWE_TLR, and Hyogen groups (Figure 2C).

At D28, the SWE_TLR and Lipo_DDA:TDB groups were significantly higher than the control group for the percentage of CD4^+^ TNF^+^IFN-γ^+^ T cells (Figure 2D) and the PLGA_TLR group was significantly higher than the control group for the percentage of CD4^+^IL17A^+^ (Th17) cells (Figure 2E). For the percentage of CD8^+^ TNF^+^IFN-γ^+^ T cells, groups Lipo_AMP and lipo_DDA:TDB were significantly higher compared to the control animals (Figure 2D). Despite the lack of statistical significance, other vaccines also appeared to have induced specific T cell immunity in some animals. For the CD4^+^ TNF^+^IFN-γ^+^ cells, three animals were above the 99% CI threshold in the Lipo_AMP group and two in the Hyogen group. For the CD8^+^ TNF^+^IFN-γ^+^ cells, two pigs were above the 99% CI threshold in the Lipo_TLR group, five in the SWE_TLR group and three in the Hyogen group (Figures 2D–F).

When focusing on TNF^−^IFN-γ^+^-producing T cells, we found a high level of non-specific responses at both D14 and D28 in the unvaccinated group which “masked” the vaccine induced responses (Supplementary Figure 2). Only Lipo_DDA:TDB induced a significant level of CD4^+^ TNF^+^IFN-γ^−^-producing T cells at D28 (Supplementary Figure 3).

In conclusion, the vaccines SWE_TLR and Lipo_DDA:TDB induced a statistically significant Th1 driven T cell response. In the groups receiving the Hyogen and Lipo_AMP formulations, despite a trend suggesting stimulation of Th1 responses, the differences were not statistically significant in the current setting. Interestingly, the PLGA_TLR formulation was the only vaccine candidate which significantly induced a Th17 response, although only 3/6 animals in this group were above the threshold.

### Blood Transcriptional Modules Correlating to Vaccine-Induced Adaptive Immune Responses

In order to shed light on the immunological perturbations associated with adaptive immune responses, changes in transcriptional modules expression were correlated to the immune responses shown in Figures 1, 2.

**Figure 3:**
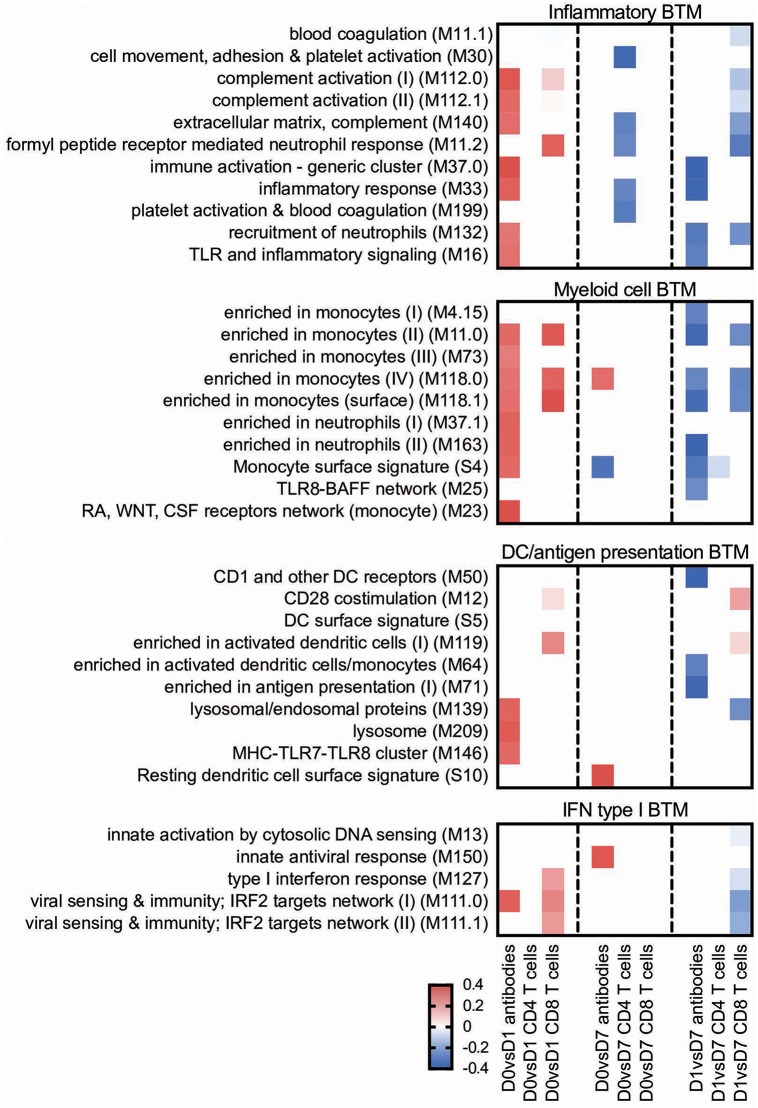
Innate immune response BTM correlating with adaptive immune responses. Time-dependent changes in scores for BTM were determined using ssGSEA and then correlated to antibodies, *M. hyopneumoniae*-specific INFγ^+^TNF^+^ CD4 T cells and CD8 T cells. Pearson correlation coefficients for the BTM changes from D0 to D1 (D0 vs. D1), from D0 to D7 (D0 vs. D7), and from D1 to D7 (D1 vs. D7) are shown as heat maps. A *P* < 0.05 was used as cut-off. Red colors indicate positive and blue negative correlations. The BTM were grouped into inflammatory, myeloid cell, DC/antigen presentation and IFN type I BTM as previously described (34). BTM, blood transcriptional modules.

For the early transcriptional responses (determined as modular changes between D0 and D1), a total of seven inflammatory, eight myeloid cell, three DC/antigen presentation and one IFN type I BTM correlated positively with the antibody response. Interestingly, none of these modules correlated with the CD4 T cell response, but some with the CD8 T cell response. For the late transcriptional responses (determined as modular changes between D1 and D7), a negative correlation was found for many BTM belonging to the families of modules reflecting innate immune responses. This was found again mainly for the antibody and CD8 T cell responses (Figure 3).

**Figure 4:**
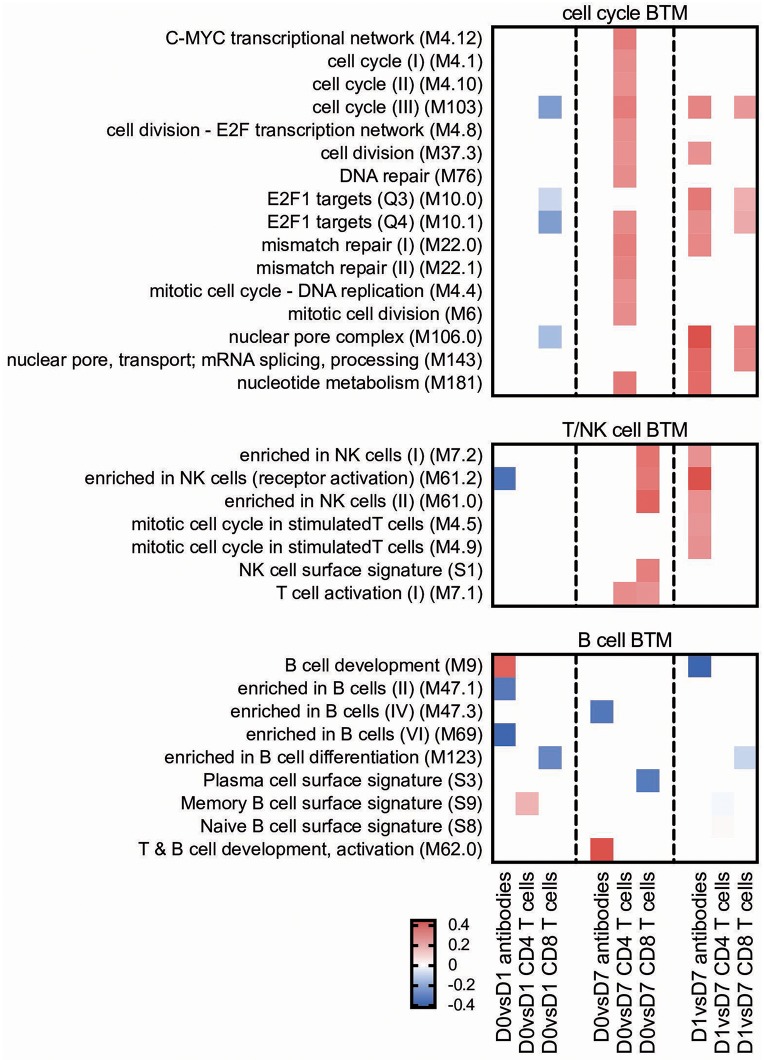
Cell cycle and lymphocyte BTM correlating with adaptive immune responses. Time-dependent changes in scores for BTM were determined using ssGSEA and then correlated to antibodies, *M. hyopneumoniae*-specific INFγ^+^TNF^+^ CD4 T cells and CD8 T cells. Pearson correlation coefficients for the BTM changes from D0 to D1 (D0 vs. D1), from D0 to D7 (D0 vs. D7) and from D1 to D7 (D1 vs. D7) are shown as heat maps. A *P* < 0.05 was used as cut-off. Red colors indicate positive and blue negative correlations. The BTM were grouped into cell cycle, T/NK cell and B cell BTM as previously described (34). BTM, blood transcriptional modules.

Main positive correlations of the CD4 T cell response were the D0 to D7 changes in cell cycle BTM (Figure 4). For the change of cell cycle BTM between D1 and D7, we also found many modules correlating with antibody and T cell responses.

T/NK cell BTM upregulation between D0 and D7 correlated well with CD8 T cell responses. The induction of these BTM also correlated with antibody levels between D1 and D7 (Figure 4).

### Transcriptional Profiling of Vaccines

**Figure 5:**
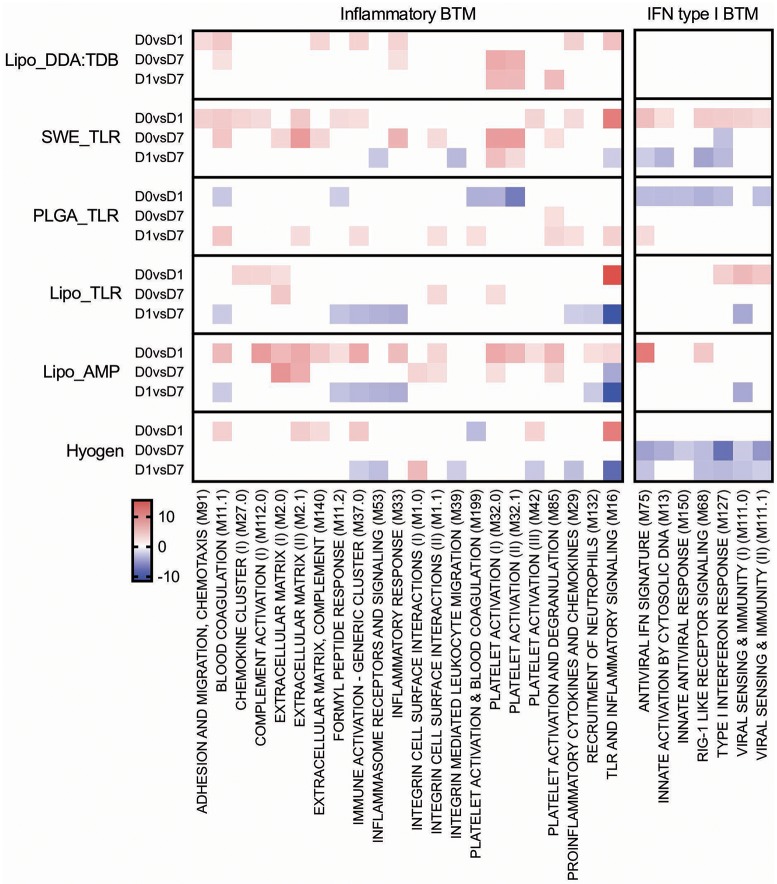
Inflammatory and IFN type I BTM induced by vaccines. The heat maps show the vaccine-dependent induction of BTM activity determined for D0 to D1 (D0 vs. D1), for D0 to D7 (D0 vs. D7), and for D1 to D7 (D1 vs. D7) changes in the modules. The values shown were calculated by −log(P-value)*1 for positively enriched BTM and as −log(P-value)* −1 for negatively enriched BTM. A cut-off of an FDR of *q* < 0.1 was employed. Red colors indicate BTM upregulation and blue downregulation. BTM, blood transcriptional modules.

**Figure 6:**
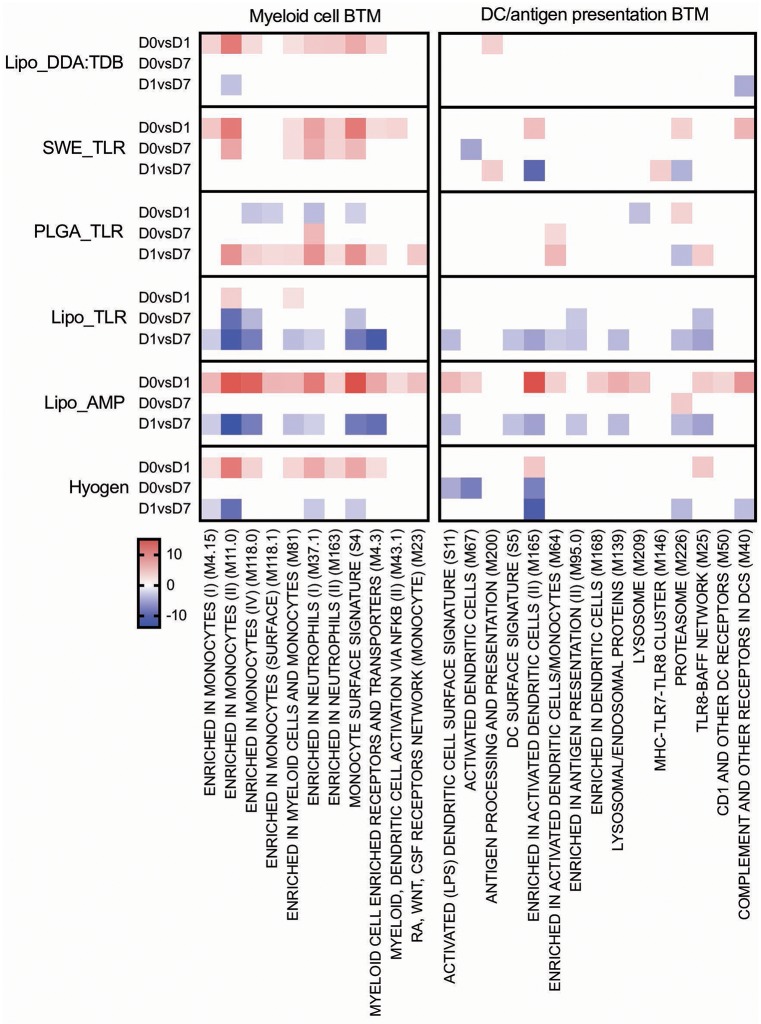
Myeloid cell and DC/antigen presentation BTM induced by vaccines. The heat maps show the vaccine-dependent induction of BTM activity determined for D0 to D1 (D0 vs. D1), for D0 to D7 (D0 vs. D7), and for D1 to D7 (D1 vs. D7) changes in the modules. The values shown were calculated by −log(P-value)^*^1 for positively enriched BTM and as −log(P-value)^*^ −1 for negatively enriched BTM. A cut-off of an FDR of *q* < 0.1 was employed. Red colors indicate BTM upregulation and blue downregulation. BTM, blood transcriptional modules.

**Figure 7:**
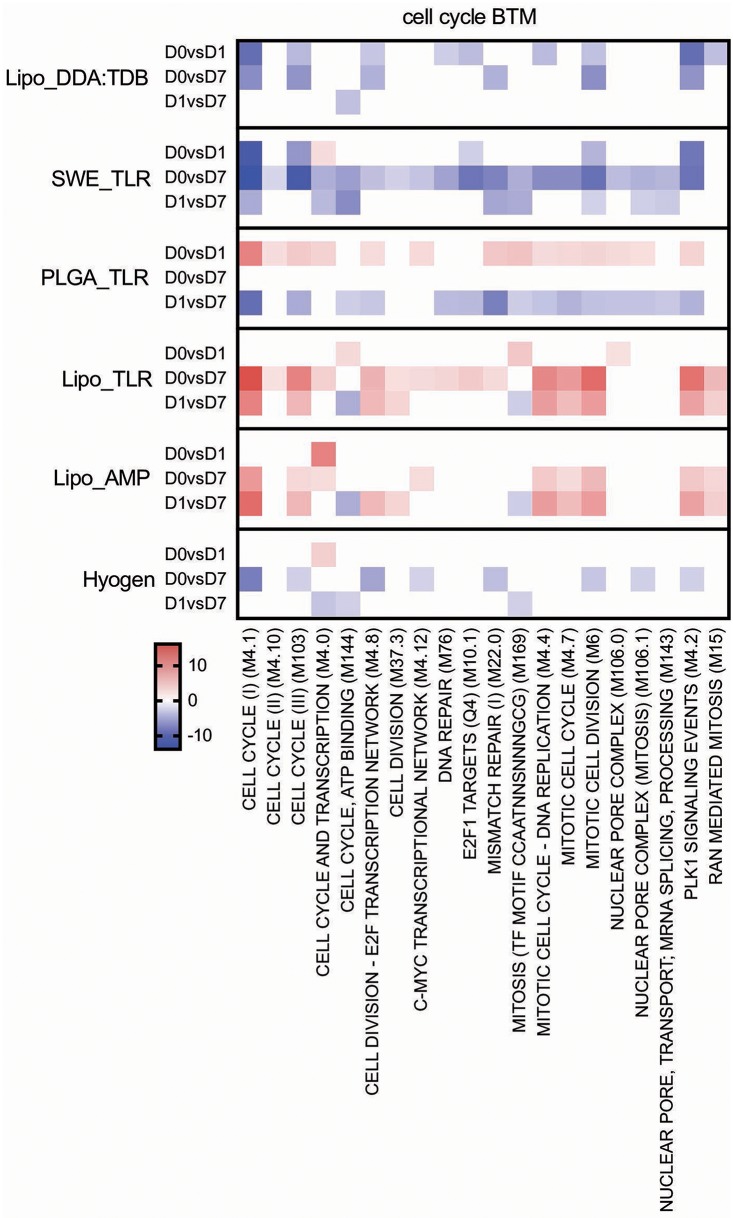
Cell cycle BTM induced by vaccines. The heat maps show the vaccine-dependent induction of BTM activity determined for D0 to D1 (D0 vs. D1), for D0 to D7 (D0 vs. D7), and for D1 to D7 (D1 vs. D7) changes in the modules. The values shown were calculated by −log(P-value)^*^1 for positively enriched BTM and as −log(P-value)^*^ −1 for negatively enriched BTM. A cut-off of an FDR of *q* < 0.1 was employed. Red colors indicate BTM upregulation and blue downregulation. BTM, blood transcriptional modules.

**Figure 8:**
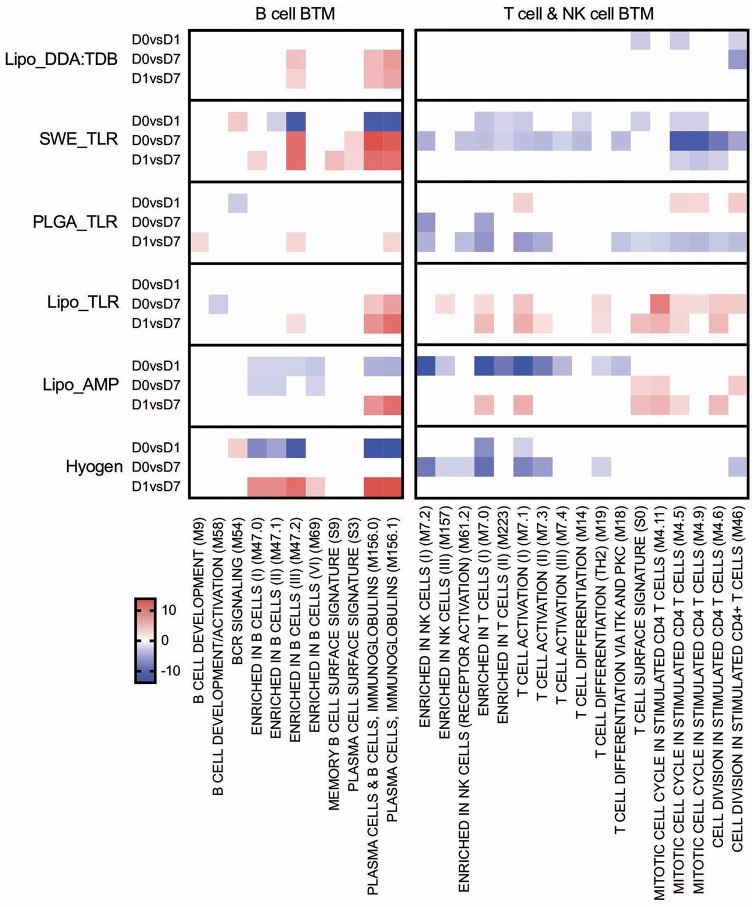
B cell and T/NK cell BTM induced by vaccines. The heat maps show the vaccine-dependent induction of BTM activity determined for D0 to D1 (D0 vs. D1), for D0 to D7 (D0 vs. D7), and for D1 to D7 (D1 vs. D7) changes in the modules. The values shown were calculated by −log(P-value)*1 for positively enriched BTM and as −log(P-value)* −1 for negatively enriched BTM. A cut-off of an FDR of *q* < 0.1 was employed. Red colors indicate BTM upregulation and blue downregulation. BTM, blood transcriptional modules.

To better understand differences in the induction of immune responses between the vaccines, we next performed a transcription profiling. From the reads obtained, we first calculated the differentially expressed genes (DEG) using DSeq2, and then employed a two-factorial model, including the factors time point and group (vaccine vs. control), to identify genes differing between two time points in vaccinated animals compared to the controls. Next, we used ranked GSEA analyses using BTM as gene sets and ranked DEG between D0 and D1, D0 and D7, and D1 and D7 of each vaccine group. All data are shown in Figures 5**–8** and Supplementary Figures 4, 5, and summarized in Table 2.

**Table 2:** Overview of the immune responses induced by the *M. hyopneumoniae* bacterins.

**Vaccine formulation**	**Ab response (D28)**	**Th1 response (D14/D28)**	**Th17 response (D14/D28)**	**Early inflam. BTM**	**Early IFN type I BTM**	**Early myeloid cell/DC BTM**	**Late cell cycle BTM**	**Late T/NK-cell BTM**	**Late Ig BTM**
Lipo_AMP	++	+	–	+++	+	+++	++	++	++
Lipo_TLR	+	+	–	+	+	+	++	++	++
PLGA_TLR	–	–	+	–	–	–	–	–	–
SWE_TLR	+	++	–	++	++	++	–	–	+++
Lipo_DDA:TDB	++	+++	–	+	–	++	–	–	++
Hyogen	++	+	–	+	–	++	–	–	++

*Six animals per group were prime-boost vaccinated on D0 and D14. Ab, antibody; Th, T helper; BTM, blood transcriptional modules; early, upregulation from D0 to D1; late, upregulation from D1 to D7; –, none to weak; +, moderate; ++, strong; +++, very strong*.

#### Inflammatory Responses

From D0 to D1, the Lipo_AMP formulation induced the highest number of inflammatory BTM, followed by the groups SWE_TLR and Lipo_DDA:TDB (Figure 5). Interestingly, in the PLGA_TLR group, no inflammatory BTM were induced and some even showed a downregulation. For the D0 to D7 comparison, again groups Lipo_AMP and SWE_TLR showed the highest upregulation of these BTM. For the D1 to D7 comparison, we found a downregulation of inflammatory modules in the Lipo_AMP, Lipo_TLR, and the Hyogen groups but not in the Lipo_DDA:TDB group, which still had BTM related to platelet activation overexpressed. In the PLGA_TLR group, eight BTM were upregulated indicating a delayed innate immune response. In summary, the three vaccines which induced significant T cell responses in terms of IFNγ/TNF secreting cells as well as antibody responses were those with the strongest positive early upregulation of inflammatory BTM, confirming the results obtained using the correlation analysis (Figure 3).

#### IFN Type I Responses

With respect to IFN type I BTM, only vaccines which contained IFN inducers such as c-di-AMP (Lipo_AMP) and CpG (Lipo_TLR, SWE_TLR) induced an early IFN type I BTM response. The PLGA_TLR formulation contained the same TLR cocktail as SWE_TLR and Lipo_TLR, but was unable to induce such responses (Figure 5).

#### Myeloid and DC/Antigen Presentation Responses

All vaccines with the exception of PLGA_TLR induced an early (D0 to D1) myeloid cell response (Figure 6). The number of BTM being modulated was the highest in the Lipo_AMP group, followed by the groups SWE_TLR, Lipo_DDA:TDB, and Hyogen (the latter two being very similar). The PLGA_TLR formulation actually had a negative influence on myeloid cell BTM response. Only Lipo_AMP, Lipo_TLR, and Hyogen induced a clear downregulation of these BTM from D1 to D7. This was interesting considering that a late D1 to D7 downregulation of myeloid cell BTM was found to strongly correlate with antibody and CD8 T cell responses (Figure 3). In summary, the vaccines which induced good adaptive immune responses were also those which induced an early induction of many myeloid cell BTM.

The Lipo_AMP formulation was found to be the most potent to induce BTM relating to DC and antigen presentation from D0 to D1. Similar to the myeloid cell BTM, DC/antigen presentation BTM were downregulated from D1 to D7 by the formulations Lipo_AMP and Lipo_TLR, and to a lower extent by the Hyogen vaccine.

#### Cell Cycle/Proliferation

The Lipo_DDA:TDB and SWE_TLR vaccines were found to downregulate, while PLGA_TLR upregulated cell cycle BTM from D0 to D1 (Figure 7). The two liposomal formulations Lipo_AMP and Lipo_TLR had a clear positive effect on these BTM at later time points (D0 to D7 and D1 to D7). Interestingly, the correlation analyses demonstrated a clear association between the late (D0 or D1 to D7) upregulation of these BTM and adaptive immune responses (Figure 4).

#### B Cell BTM and T/NK Cell BTM

The Lipo_AMP, SWE_TLR, and Hyogen vaccines had an overall negative effect on the early expression (D0 to D1) of B-cell BTM (Figure 8). The SWE_TLR and Hyogen formulations were those to strongly induce these BTM at later time points (D1 to D7). Common BTM between the strong vaccines in terms of antibody responses were plasma cells and immunoglobulin (M156.0 and M156.1), which were overexpressed from D1 to D7. However, these BTM were not found significant in the correlation analyses (Figure 4).

For the T cell/NK cell BTM, a variable early downregulation by the more immunogenic vaccines Lipo_AMP, SWE_TLR, Lipo_DDA:TDB, and Hyogen was found. Only the liposomal formulations Lipo_AMP and Lipo_TLR induced a late D0/D1 to D7 upregulation of these modules, although many of those modules correlated to antibody and T cell responses (Figure 4).

## Discussion

The present study assessed the safety and performed a detailed immunological profiling of five novel *M. hyopneumoniae* bacterin formulations. We included as well the commercial vaccine Hyogen® in our study. Hyogen® is a recently developed bacterin based on a virulent *M. hyopneumoniae* field isolate with a TLR4 ligand as immunostimulant, and in that way comparable with our experimental bacterin formulations.

In terms of side effects, formulations Lipo_AMP, Lipo_TLR, and SWE_TLR induced a significant but transient increase in rectal body temperature shortly (4 h) after vaccination. This was also observed for the Hyogen® vaccine, and comparable observations were made by Llopart et al. (50) after two shot vaccination against *M. hyopneumoniae* with the commercial vaccine Mypravac suis® (HIPRA, Amer, Spain). Fever beginning a few hours after vaccination and persisting for 24 to 36 h is the result of an excessive induction of pro-inflammatory cytokines by the vaccine (51, 52). Systemic reactions of such kind are commonly reported and considered as “normal toxicity” associated with vaccination (52).

Overall, the ISR occasionally observed in all IM vaccinated groups and at the IM injection site of group Lipo_DDA:TDB were mild and resolved quickly. Transient redness and swelling at vaccination sites were also reported in other *M. hyopneumoniae* vaccination studies (53–55). Such local reactions often occur after parenteral administration of adjuvanted vaccines and are tolerated in terms of safety (52). Microscopically, mild focal chronic inflammation was observed in all IM injected groups, including the control group, indicating that these findings were probably caused by the tissue damage due to needle insertion and injection of fluid, and not by the administered vaccine formulation. Nevertheless, prolonged mild to moderate ISR were observed in all pigs from group Lipo_DDA:TDB at the ID injection site and histopathological examination of this injection site at D28 of the study showed a severe foreign body granuloma in five pigs. Local reactions of such kind could result in carcass trim losses at slaughter and are therefore considered to be a relevant adverse side effect of vaccination (51, 56). The transient ISR at the IM injection site of this vaccine group suggests that the prolonged and rather severe ISR is at least partially due to the ID administration. However, this cannot be stated with certainty as there was no control group ID injected with sterile PBS.

Two weeks after booster vaccination, the commercial vaccine Hyogen® as well as the vaccines Lipo_DDA:TDB and Lipo_AMP induced a strong humoral response. Vaccine formulations SWE_TLR and Lipo_TLR generated a moderate serological response, whereas for the PLGA_TLR formulation only two animals seroconverted. Nevertheless, as we do not know the antigen payload of the Hyogen® vaccine, we cannot directly compare its efficacy to the experimental vaccines. Although systemic antibodies are considered to play a minor role in protection against EP (5, 13), high levels of serum antibodies induced by vaccination can be an easy and practical tool to confirm successful vaccination in the field (57). It can also be expected that high levels of IgG will only be induced with significant induction of Th cell activation. We only found lgA antibodies in BAL fluid of one pig injected with the SWE_TLR vaccine. This is not surprising considering the parenteral vaccine administration, and is in line with previous studies showing IgA in BAL fluid of vaccinated pigs only after challenge (13, 14). Future studies are required to investigate the potential of adjuvants to induce both local and systemic immune responses after mucosal application of the vaccine. For example, this has been achieved for inactivated viruses using nanoparticle-based delivery (58, 59). Nevertheless, the absence of detectable IgA antibodies in BAL fluid from vaccinated pigs does not exclude priming of the immune system for such responses as vaccinated animals had higher mucosal IgA responses compared to unvaccinated animals following challenge (13, 14). Although we did not measure *M. hyopneumoniae-*specific IgG in BAL fluid, it can reach the alveolar lumen by transudation from the blood and might also play a role in protection against disease. In fact, the implementation of the human parenterally-administered conjugate vaccine against type B *Haemophilus influenza* resulted in a reduction of carriage and a reduced risk of horizontal transmission. This was hypothesized to be due to such IgG (60, 61).

Circulating *M. hyopneumoniae*-specific TNF^+^IFN-γ^+^ CD4 and CD8 T cells were identified in particular in the SWE_TLR, Lipo_DDA:TDB, and Lipo_AMP groups. However, also in the Lipo_TLR and Hyogen groups a few animals appeared to have such cells. Such Th1 response is expected to promote cell-mediated immunity via activation of NK cells and macrophages, as well as by inducing antigen-specific cytotoxic immunity (CD8 cells) (62). While such responses could participate in protection against *M. hyopneumoniae*, pro-inflammatory CD4 Th responses might also mediate lung damage and clinical disease (63). While the classical effector functions of CD8 T cells are likely irrelevant for the immune response against a *Mycoplasma* species that is not an intracellular organism, mouse models indicate that CD8 T cells are suspected to dampen inflammatory responses mediated by CD4^+^ Th cells (24, 64). Furthermore, CD8 T cells contribute to Th1 responses, which based on mouse models could be protective against *Mycoplasma* infection. Based on this their induction by the present vaccines could be viewed as positive.

In this study, the PLGA_TLR formulation was the best at inducing a Th17 response, although in only three of six animals a response was detected. The lack of detection of IL-17-producing Th cells does not mean a lack of priming, as activated/memory T cells could have left the blood circulation. Nevertheless, future studies are required to confirm the ability of this formulation to induce Th17 responses. It has been suggested that Th17 cells may play a major role in the protection of the lung mucosa against respiratory pathogens by recruiting other immune cells to the inflamed mucosa for pathogen clearance (65) and by promoting IgA secretion into the airway lumen (66). Similar to other species, porcine IL-17-producing cell differentiation can be induced *in vitro* by TGF-β in the presence of IL-6 and/or IL-1β (67), and *in vivo* during several extracellular bacterial infections (68–70).

In addition to these classical vaccinology readouts we also applied a transcriptomics-based approach to obtain a more precise profile of the type of immune response induced by our vaccine candidates, and to better understand the immune modulatory effector functions needed for induction of a protective immune response after vaccination. We identified a number of BTM correlating to adaptive immune responses which have been previously reported in human and sheep studies (25, 26, 28–30, 34). This was an early upregulation of monocytes BTM, such as S4, M11.0, M118.0, and M118.1, neutrophil BTM such as M163 and M37.1, modules related to inflammation and pathogen sensing such as M16, M146, and M37.0, and BTM related to antigen presentation such as M147, M139, and M209. Interestingly, many of these modules strongly negatively correlated with the antibody and CD8 T cell responses from D1 to D7. This suggests that a strong innate immune response in the first 24 h followed immediately by a down-regulation is associated with the initiation of a stronger adaptive immune response. This inverse correlation was also seen at later time points in a previous study using sheep (34). Similar to previous reports (25, 28, 29, 34) a few cell cycle, B cell, and T/NK cell BTM upregulated in the first 24 h after vaccination negatively correlated with adaptive immune responses. While these correlations were mainly found for the antibody responses, most of the modules correlating to CD4 T cell responses were found to be cell cycle BTM upregulated between D0 and D7. This was also reported by Qi et al. (30) who found a strong association of cell cycle and DNA repair BTM to virus-specific T cells (common BTM are M4.4, M4.12, M103, M76, M22.0, M22.1). The upregulation of T/NK cell modules between D0 and D7 positively correlated to CD8 T cell responses, as well as to the antibody responses between D1 and D7. Altogether, these results confirm the importance of early innate immune responses in the myeloid and DC cell compartment within the first 24 h for a potent vaccine-induced adaptive immune response. Clearly, the upregulation of myeloid cell and DC/antigen presentation BTM could partially reflect changes in cell population, i.e., those that are caused by enhanced hematopoiesis following stimulation of the innate immune system (71). Nevertheless, in a previous study we were unable to identify a significant increase in the circulation of monocytes, indicating that the BTM changes reflect more than changes in cell populations (34). This study also demonstrates that the main upregulated BTM from D0 or D1 to D7 correlating to adaptive immune responses are cell cycle and T/NK cell BTM. This could reflect the first recirculation of activated T cells leaving the lymph nodes that drain the site of vaccine injection.

After obtaining this information, we went back and analyzed which BTM were actually induced by the vaccines. While all vaccines, with the exception of the PLGA_TLR formulation, induced early upregulation of inflammatory, myeloid cell and DC/antigen presentation BTM, the Lipo_AMP vaccine appeared to be the most potent in stimulating these early innate immune responses. When it came to the later upregulation of cell cycle and T/NK cell BTM, this was only a feature of the Lipo_AMP and the Lipo_TLR vaccines. This was surprising considering that the Lipo_TLR was not found to be a particularly potent formulation. Furthermore, the more potent vaccines, such as Lipo_DDA:TDB and SWE_TLR, actually induced a downregulation of these BTM. While this requires further investigations, our current interpretation is that there could be differences in the kinetics of activated lymphocyte recirculation, which would have required more frequent sampling to detect. Furthermore, it should again be noted that T cell recirculation and the presence of memory T cells in the circulation is a dynamic process. Therefore, a lack of antigen-specific T cells in the peripheral blood cannot be interpreted as a lack of priming. On the other hand, the BTM profile induced by the PLGA_TLR vaccine was in line with its rather poor immunogenicity.

Overall, our data demonstrate the potency of cationic liposome formulations as delivery system to induce potent B and T cell responses using inactivated *M. hyopneumoniae* as antigen. Cationic liposomes may have the advantage of a more targeted delivery of the immunostimulant and antigen to DC, and also have been shown to enhance the retention time in lymph nodes (72, 73). This may favor strong T cell responses. Although we did not specifically address the requirement of an immunostimulant for liposomal vaccines, it is well-described that immunogenicity of liposomal vaccines can be enhanced (72). Our data indicate that both AMP and TDB appear to be good candidate molecules for the *M. hyopneumoniae* vaccine. The transcriptomic profile of the Lipo_AMP vaccine was particularly impressive as it corresponded best to a BTM profile known to correlate with adaptive immune responses. From all experimental formulations, Lipo_DDA:TDB induced the highest antibody and Th1 responses. Unfortunately, this formulation caused a prolonged ISR after ID administration. Applying this vaccine only via the IM route could resolve this safety issue, but it would be probably associated with a loss of immunogenicity (74). In contrast, the PLGA-based MP formulation did not appear to be suitable to induce good antibody and Th1 responses, possibly in part due to a delayed TLR ligand delivery to innate immune cells. Nevertheless, the fact that this vaccine induced IL17-producing Th cells at least in some animals is interesting and should be kept in mind for future investigations. The present work also identified the SWE_TLR as an interesting vaccine candidate, as it induced a robust Th1 response and IgA in BAL fluid of one animal. This vaccine has the advantage of being easy to produce. Moreover, O/W formulations are known to have a much better safety profile as W/O vaccines (72). Future studies are required to address which immunostimulant is best suited for a SWE adjuvant in the pig. This will require the use of a selection of identical antigens.

In conclusion, the present study identified promising *M. hyopneumoniae* bacterin formulations to be selected for future challenge experiments, based on their ability to induce strong innate immune responses and robust Th1 or Th17 responses. We also demonstrated the utility of transcriptome-based systems immunology analyses to unravel the mechanistic events leading to the stimulation of adaptive immune responses after vaccine injection. While the present study was not designed to identify the effects of formulation and immunostimulants but rather to select the most promising candidates from five novel vaccines, the information provided on these vaccine formulations will also be very valuable for other vaccines and future adjuvant research.

## Ethics Statement

This study was carried out in accordance with the following laws and directives on animal experimentation in Belgium: ‘KB 29/05/2013 betreffende de bescherming van proefdieren, KB 31/12/2012 herziening wet 86, ETS 123 richtlijnen huisvesting proefdieren' and the EU 2010/63 directive on the protection of animals used for scientific purposes. The protocol was approved by the Ethical Committee for Animal Experiments of the Faculty of Veterinary Medicine, Ghent University (approval number EC2016/91).

## Author Contributions

AMM, GA, OG-N, ROB, BD, and AM performed the animal experimentation, acquisition and analyses of data. VJ, CG, CB-Q, and NC designed, produced, and characterized the vaccines. IK, RB, and AS performed the bioinformatic analyses. DM, CB-Q, FB, FH, and AS designed and supervised the overall project.

### Conflict of Interest Statement

CG is named as inventor in a patent covering the use of c-di-AMP as adjuvant (PCT/EP 2006010693). The remaining authors declare that the research was conducted in the absence of any commercial or financial relationships that could be construed as a potential conflict of interest.

## Footnotes

^1^http://www.bioinformatics.babraham.ac.uk/projects/fastqc/

^2^www.ebi.ac.uk/ena

^3^http://software.broadinstitute.org/cancer/software/genepattern/modules/docs/ssGSEAProjection/4

## Supplementary Material

Data Sheet 1 List of genes for each BTM.

## Abbreviations

EP: enzootic pneumonia
CCU: color changing units
PLGA: poly(lactic-co-glycolic acid)
SWE: squalene-in-water emulsion
DDA: dimethyl dioctadecylammonium
DPPC:DC-Chol, 1: 2-dipalmitoyl-sn-glycero-3-phosphocholine and dimethylaminoethane carbamoyl cholesterol
TDB, trehalose 6: 6-dibehenate
c-di-AMP: cyclic diadenylate monophosphate
PAM: Pam3Cys-SK4
CpG: CpG ODN SL03
PS: particle size
Pdi: polydispersity index
ZP: zeta potential
IM: intramuscularly
ID: intradermally
ISR: injection site reaction
ADG: average daily gain
BAL: bronchoalveolar lavage
GSEA: gene set enrichment analysis
FDR: false discovery rate
DEG: differentially expressed genes
BTM: blood transcriptional modules.
