# Supplementary material for: Systems Immunology Characterization of Novel Vaccine Formulations for Mycoplasma hyopneumoniae Bacterins
Source: Front Immunol. 2019 May 24;10:1087. doi: 10.3389/fimmu.2019.01087 (PMC6543460; doi:10.3389/fimmu.2019.01087)
Supplement: Data Sheet 1 — List of genes for each BTM. [file Data_Sheet_2.pdf]

Supplementary Tables and Figures

Supplementary Table 1. Physicochemical characterization of the adjuvants. DPPC:DC-Chol, 1,2-dipalmitoyl-sn-glycero-3-phosphocholine and dimethylaminoethane carbamoyl cholesterol; c-di-AMP, bis-(3',5')-cyclic dimeric adenosine monophosphate; PLGA, poly(lactic-co-glycolic acid) and ethylaminoethyl-dextran; DDA, dimethyl dioctadecylammonium; TDB, trehalose 6,6'-dibehenate; dna, data not available; Pdi, polydispersity; µm, micrometers; nm, nanometers; mV, millivolts.

Adjuvants	Particle size	Polydispersity	Zeta potential (mV)	Immune-stimulators (loading ratio)
Lipo_AMP	134 nm	Pdi = 0.03	+ 52.6	C-di.AMP (67 %)
Lipo_TLR	132 nm	Pdi = 0.08	+ 45.2	Pam3Cys-SK4 / CpG ODN SL03 / Resiquimod (98% / 80% / dna)
PLGA_TLR	9.25 µm	Span = 1.16	+17.8	dna
SWE_TLR	145 nm	Pdi = 0.06	- 31.13	mixed
Lipo_DDA:TDB	133 nm	Pdi = 0.07	+ 50.7	dna

Supplementary Table 2. Scoring system used for the evaluation of intramuscular and intradermal injection site reactions. Adapted from a score system kindly provided by CEVA Santé Animale, Libourne Cedex, France. IM, intramuscular; ID, intradermal.

Score		Description
**0**	Normal	no more than a visible injection site<br>*IM: less than about 0,5 diameter zone of redness surrounding injection site*<br>*ID: cutaneous swelling only associated with the injection site*
**1**	Mild	0.5-2 cm diameter<br>discoloration<br>no distinct palpable swelling<br>may be irritation (occasional rubbing at injection site)
**2**	Moderate	2-5 cm diameter<br>discoloration<br>(or) palpable swelling<br>may be irritation (persistent rubbing at injection site)
**3**	Severe	>5 cm diameter<br>discoloration<br>(and) visible and palpable swelling<br>irritation and pain (persistent rubbing at injection site, withdrawal and vocalization upon palpation<br>may be abscess, exudate

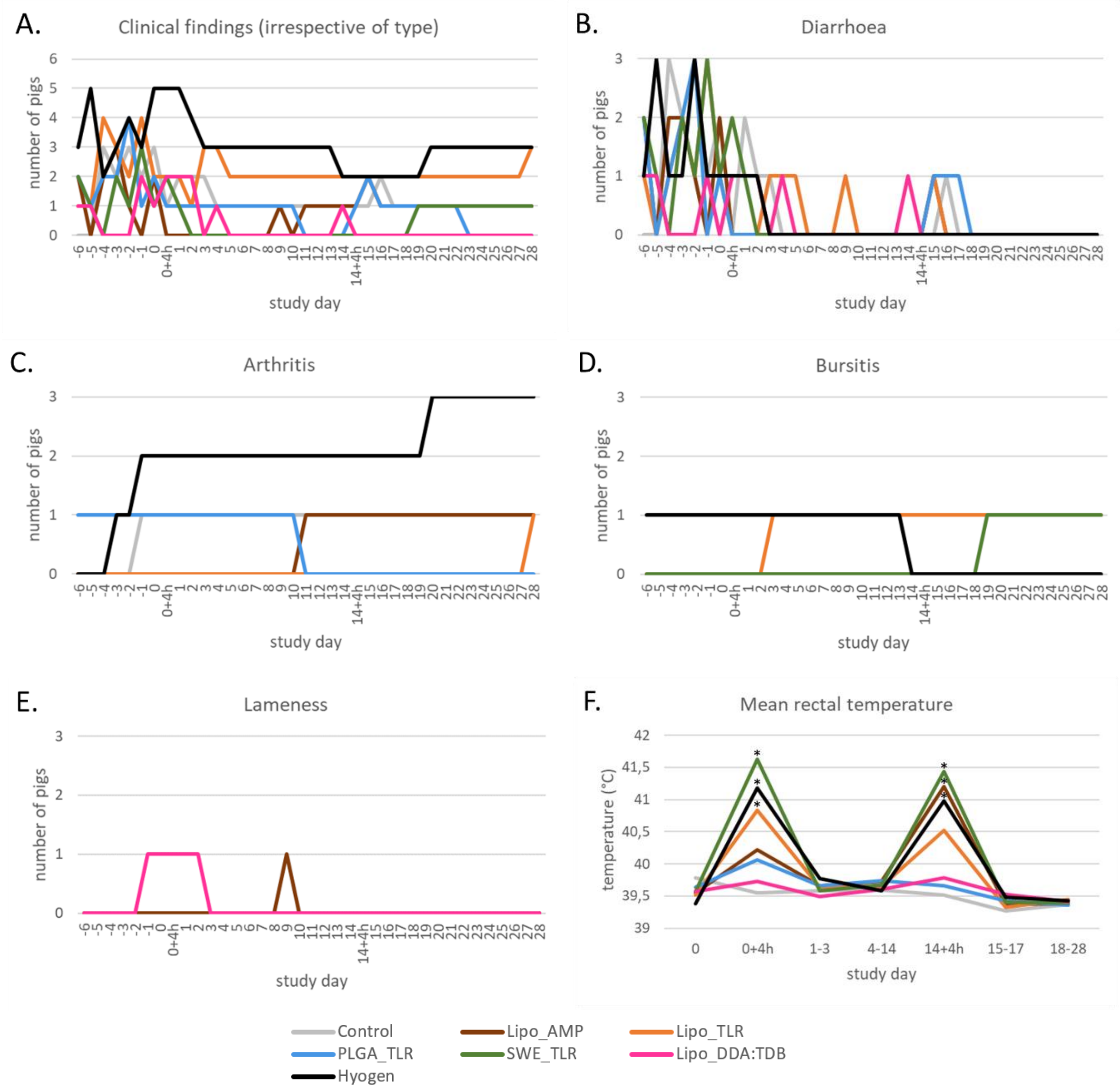

Supplementary Figure 1. General health and mean rectal temperature of the piglets during the whole study period. Six animals per group were vaccinated on D0 and D14. For the parameter rectal temperature Mann-Whitney U tests with a Bonferroni correction were used to analyze differences between the control and vaccinated groups at the different time-points (**P* < 0.05; ***P* < 0.01; ****P* < 0.001).

Supplementary Table 3. Overview of the number of pigs with ISR, duration of the ISR and severity. Six animals per group were vaccinated on D0 and D14. Significant differences in the number of animals with ISR compared to the control group were calculated using Fisher's exact tests with a Bonferroni correction (**P* < 0.05; ***P* < 0.01; ****P* < 0.001; ^{ns}not significantly different; ^{0}statistical result not computed). ISR, injection site reaction; IM, intramuscular; ID, intradermal.

Group (n)	N° of pigs		ISR duration	ISR score (description)
Control (6)	**0**			
Lipo_AMP (6)	**2^{ns}**	1	D0 + 4h	moderate (palpable swelling 0.5-1 cm Ø)
			D15	mild (redness 1 cm Ø)
		1	D14 + 4h	moderate (palpable swelling 0.5 cm Ø)
Lipo_TLR (6)	**2^{ns}**		D15	mild (redness 0.5-1 cm Ø)
PLGA_TLR (6)	**0^{0}**			
SWE_TLR (6)	**1^{ns}**		D0 + 4h	mild (redness 0.5-2 cm Ø)
Lipo_DDA:TDB IM (6)	**3^{ns}**	1	D14 + 4h until D16	moderate (palpable swelling often with redness 0.5-2 cm Ø)
		1	D15	moderate (palpable swelling 0.5 cm Ø)
		1	D15 until D16	moderate (palpable swelling (with redness) 0.5 cm Ø)
Lipo_DDA:TDB ID (6)	**6***	6	D0 + 4h until D2-5	moderate (palpable swelling often with redness 0.5-2 cm Ø)
		5	D3-6 until D4-14 + 4h	mild (little crust and/or redness)
		4	D13-24 until D28	moderate (palpable swelling often with redness 0.5-2 cm Ø)
Hyogen (6)	**2^{ns}**	1	D15	mild (redness 0.5-1 cm Ø)
			D16 until D20	severe (palpable and visible swelling > 5cm Ø)
			D21	moderate (palpable swelling)
		1	D16 until D17	mild (redness 0.5 cm Ø)

Supplementary Table 4. Results of the histopathological examination of the injection sites at D28 of the study. Six animals per group were vaccinated on D0 and D14. Significant differences in the number of animals with histopathological findings compared to the control group were calculated using Fisher's exact tests with a Bonferroni correction (*P < 0.05; **P < 0.01; ***P < 0.001; ns, not significantly different). IM, intramuscular; ID, intradermal.

Group	Control IM	Control ID	Lipo_AMP	Lipo_TLR	PLGA_TLR	SWE_TLR	Lipo_DDA:TDB	Hyogen
Number of animals with histopathological findings at the injection site								
	2	0	3^{ns}	6^{ns}	6^{ns}	6^{ns}	IM 4^{ns} ID 5^{ns}	5^{ns}
Number of different types of histopathological findings at the injection site								
**Haemorrhage**	2	0	2	1	3	3	IM 1 ID 0	1
**Blood resorption**	0	0	0	1	3	1	IM 0 ID 0	2
**Necrosis**	1	0	0	0	0	1	IM 1 ID 0	0
**Acute inflammation**	0	0	0	0	1	0	IM 0 ID 0	0
**Chronic inflammation**	2	0	1	5	3	5	IM 3 ID 5	4
**Angiogenesis**	0	0	0	0	0	0	IM 0 ID 3	0
**Proliferation of connective tissue**	0	0	0	1	0	2	IM 1 ID 4	0
Number of overall scores for histopathological findings at the injection site								
**Not detected**	4	6	3	0	0	0	IM 2 ID 1	1
**Mild**	2	0	3	6	4	5	IM 4 ID 0	5
**Moderate**	0	0	0	0	2	1	IM 0 ID 1	0
**Severe**	0	0	0	0	0	0	IM 0 ID 4	0

Supplementary Table 5. Results of the *M. hyopneumoniae* specific antibodies measured at different time points in serum and in BAL fluid. Six animals per group were vaccinated on D0 and D14. *M. hyopneumoniae* specific antibodies were determined by the IDEIA™ *Mycoplasma hyopneumoniae* EIA kit (Oxoid Limited, Hampshire, UK) and by indirect in-house ELISA's. For the in-house ELISA's, NetOD-values were calculated by subtracting the OD-value of the blank from the OD-value of the sample. BAL, bronchoalveolar lavage; OD, optical density; SD, standard deviation.

Group	Control	Lipo_AMP	Lipo_TLR	PLGA_TLR	SWE_TLR	Lipo_DDA:TDB	Hyogen
Percentage of *M. hyopneumoniae* seropositive pigs (n positive pigs/total n pigs) determined with the IDEIA™ kit from Oxoid							
**D0**	0.00 (0/6)	0.00 (0/6)	0.00 (0/6)	0.00 (0/6)	0.00 (0/6)	0.00 (0/6)	0.00 (0/6)
**D7**	0.00 (0/6)	0.00 (0/6)	0.00 (0/6)	0.00 (0/6)	0.00 (0/6)	0.00 (0/6)	0.00 (0/6)
**D14**	0.00 (0/6)	0.00 (0/6)	0.00 (0/6)	0.00 (0/6)	0.00 (0/6)	0.00 (0/6)	0.00 (0/6)
**D28**	0.00 (0/6)	100.00 (6/6)	100.00 (6/6)	33.33 (2/6)	100.00 (6/6)	100.00 (6/6)	100.00 (6/6)
M. hyopneumoniae specific antibodies in serum measured with the IDEIA™ kit from Oxoid (OD-values ± SD)							
**D0**	1.479 ± 0.176	1.490 ± 0.112	1.434 ± 0.199	1.454 ± 0.118	1.448 ± 0.217	1.465 ± 0.174	1.538 ± 0.149
**D7**	1.453 ± 0.073	1.455 ± 0.160	1.425 ± 0.071	1.517 ± 0.150	1.284 ± 0.073	1.471 ± 0.061	1.497 ± 0.149
**D14**	1.482 ± 0.113	1.149 ± 0.096	1.228 ± 0.133	1.441 ± 0.139	1.430 ± 0.108	1.245 ± 0.120	1.219 ± 0.088
**D28**	1.752 ± 0.096	0.325 ± 0.169	0.364 ± 0.151	0.837 ± 0.405	0.436 ± 0.092	0.245 ± 0.092	0.220 ± 0.115
M. hyopneumoniae specific IgG antibodies in serum measured with an indirect in house ELISA (NetOD-values ± SD)							
**D0**	0.031 ± 0.014	0.061 ± 0.059	0.033 ± 0.007	0.027 ± 0.011	0.041 ± 0.029	0.061 ± 0.046	0.035 ± 0.034
**D7**	0.049 ± 0.020	0.065 ± 0.051	0.050 ± 0.016	0.041 ± 0.009	0.065 ± 0.036	0.073 ± 0.052	0.058 ± 0.028
**D14**	0.041 ± 0.029	0.092 ± 0.046	0.062 ± 0.030	0.027 ± 0.007	0.042 ± 0.026	0.053 ± 0.036	0.030 ± 0.020
**D28**	0.055 ± 0.028	0.573 ± 0.197	0.255 ± 0.119	0.101 ± 0.115	0.172 ± 0.041	0.433 ± 0.226	0.535 ± 0.250
M. hyopneumoniae specific IgA antibodies in serum measured with an indirect in-house ELISA (NetOD-values ± SD)							
**D0**	0.006 ± 0.004	0.008 ± 0.007	0.007 ± 0.006	0.005 ± 0.002	0.007 ± 0.007	0.009 ± 0.008	0.006 ± 0.004
**D7**	0.015 ± 0.007	0.011 ± 0.006	0.017 ± 0.012	0.011 ± 0.008	0.011 ± 0.007	0.020 ± 0.017	0.012 ± 0.007
**D14**	0.019 ± 0.013	0.024 ± 0.016	0.033 ± 0.036	0.015 ± 0.007	0.022 ± 0.022	0.019 ± 0.013	0.012 ± 0.002
**D28**	0.038 ± 0.027	0.045 ± 0.017	0.030 ± 0.010	0.021 ± 0.012	0.026 ± 0.013	0.041 ± 0.018	0.055 ± 0.025
M. hyopneumoniae specific IgA antibodies in BAL fluid measured with an indirect in house ELISA (NetOD-values ± SD)							
**D28**	0.014 ± 0.010	0.024 ± 0.015	0.021 ± 0.008	0.025 ± 0.022	0.041 ± 0.045	0.027 ± 0.017	0.020 ± 0.011
Percentage of *M. hyopneumoniae* specific IgA positive pigs in BAL fluid determined with an indirect in house ELISA (n positive pigs/total n pigs)							
**D28**	0.00 (0/6)	0.00 (0/6)	0.00 (0/6)	0.00 (0/6)	16.67 (1/6)	0.00 (0/6)	0.00 (0/6)

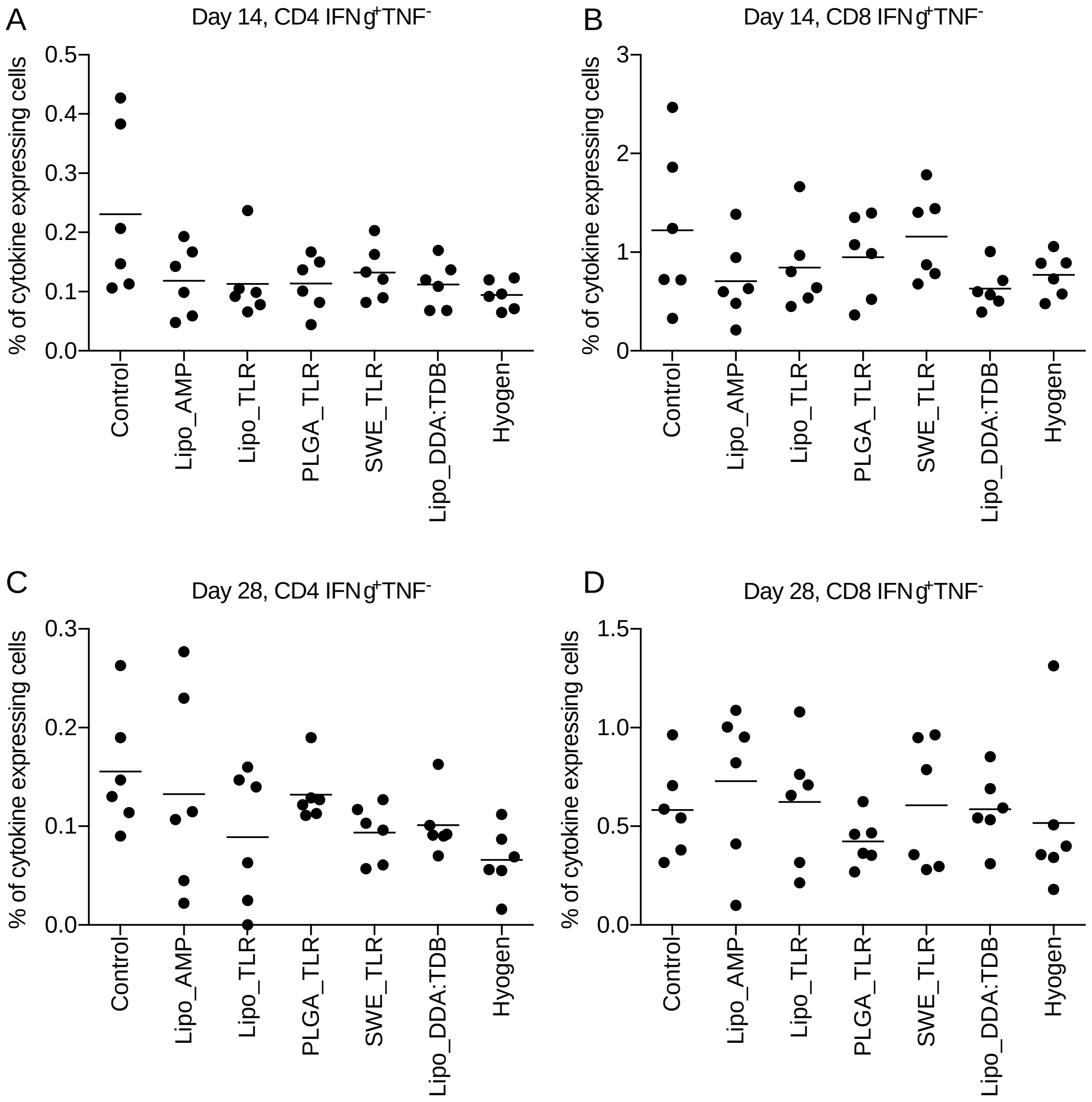

Supplementary Figure 2. ***M. hyopneumoniae*-specific IFNγ^{+}TNF^{-} T cells frequencies following vaccination of pigs with vaccine candidates**. Six animals per group were prime-boost vaccinated on D0 and D14. At D14 and D28, *M. hyopneumoniae*-specific T cells induced by the tested vaccines listed in the legend were determined by *in vitro* restimulation of PBMC from vaccinated animals followed by intracellular cytokine staining and multicolor flow cytometry. Following doublet exclusion, live cells were gated and the percentage of IFNγ^{+}TNF^{-} single positive CD4^{+} (A, C) and CD8β^{+} (B, D) T cells was determined. The mean values obtained from triplicate cultures for individual animals are shown. Significance was calculated using two-way ANOVA followed by Dunnett‘s test (**P* < 0.05; ***P* < 0.01; ****P* < 0.001). PBMC, peripheral blood mononuclear cells.

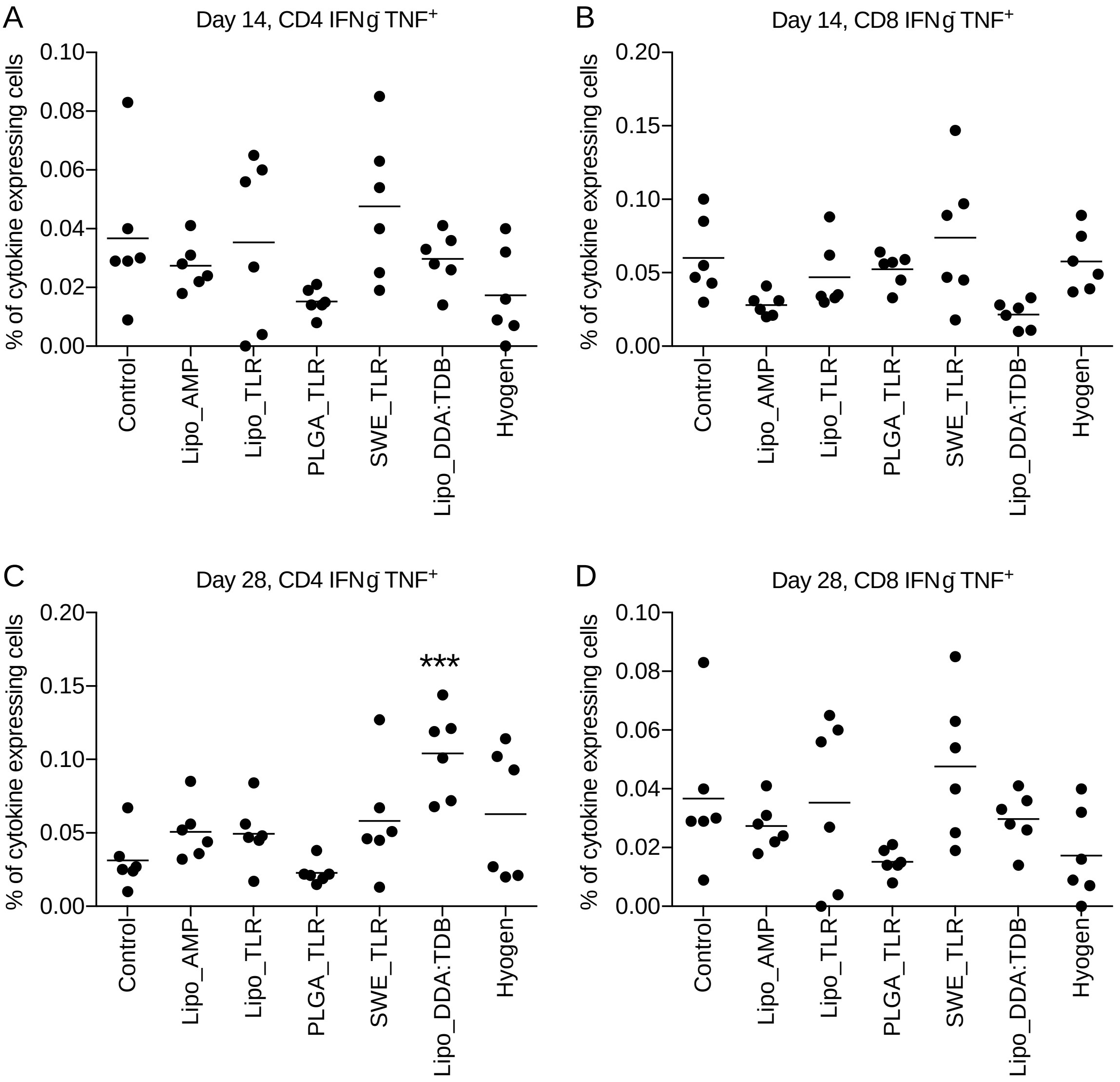

Supplementary Figure 3. ***M. hyopneumoniae*-specific IFNγ⁻TNF⁺ T cells frequencies following vaccination of pigs with vaccine candidates**. Six animals per group were prime-boost vaccinated on D0 and D14. At D14 and D28, *M. hyopneumoniae*-specific T cells induced by the tested vaccines listed in the legend were determined by *in vitro* restimulation of PBMC from vaccinated animals followed by intracellular cytokine staining and multicolor flow cytometry. Following doublet exclusion, live cells were gated and the percentage of $IFN\gamma^{-}TNF^{+}$ single positive $CD4^{+}$ (A, C) and $CD8\beta^{+}$ T (B, D) cells was determined. The mean values obtained from triplicate cultures for individual animals are shown. Significance was calculated using two-way ANOVA followed by Dunnett's test (*$P < 0.05$; **$P < 0.01$; ***$P < 0.001$). PBMC, peripheral blood mononuclear cells.

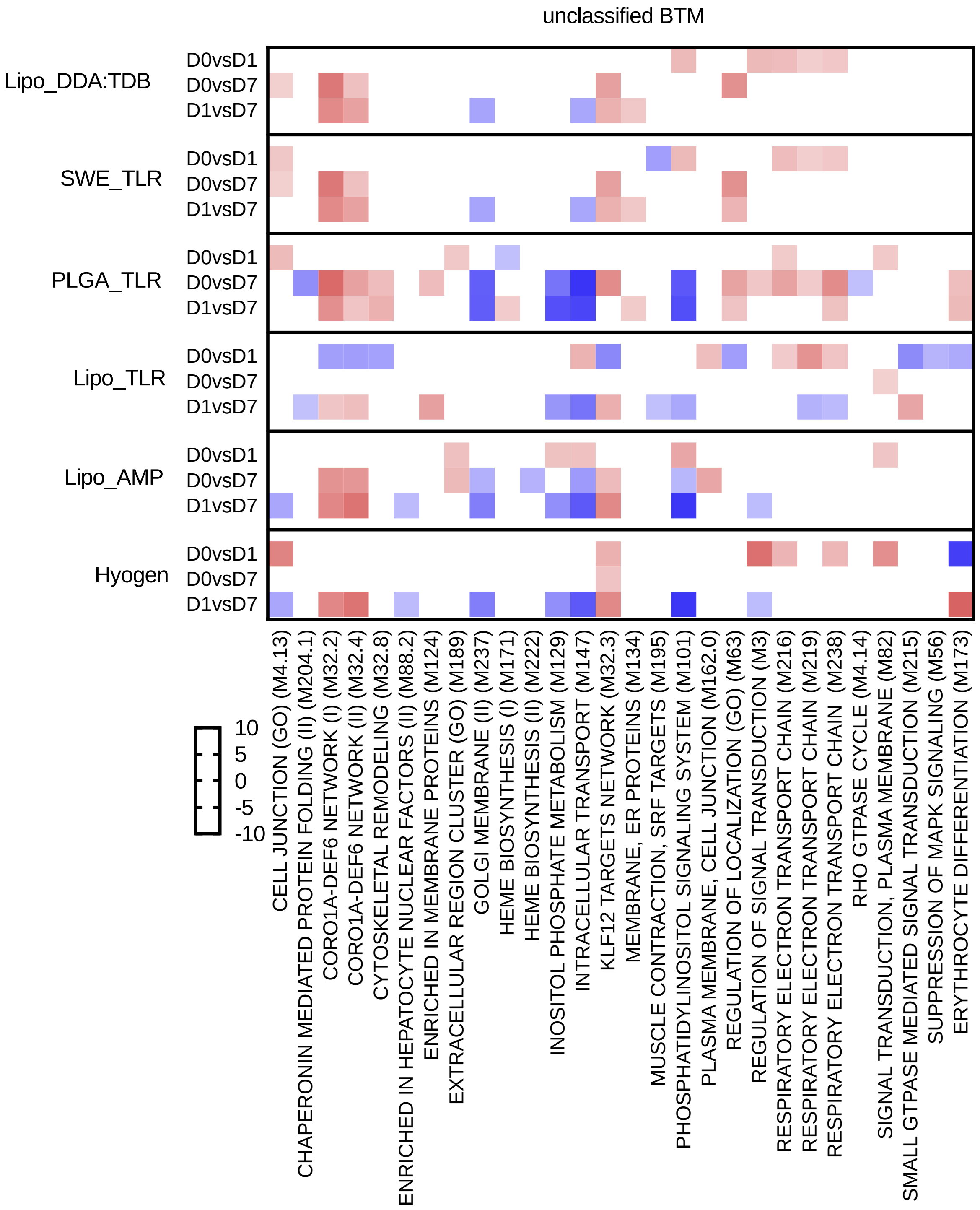

Supplementary Figure 4. Unclassified BTM induced by vaccines. The heat maps show the vaccine-dependent induction of BTM activity determined for D0 to D1 (D0vsD1), for D0 to D7 (D0vsD7) and for D1 to D7 (D1vsD7) changes in the modules. The values shown were calculated by -log(P-value)*1 for positively enriched BTM and as -log(P-value)*-1 for negatively enriched BTM. A cut-off of an FDR of q < 0.1 was employed. Red colors indicate BTM upregulation and blue downregulation. BTM, blood transcriptional modules.

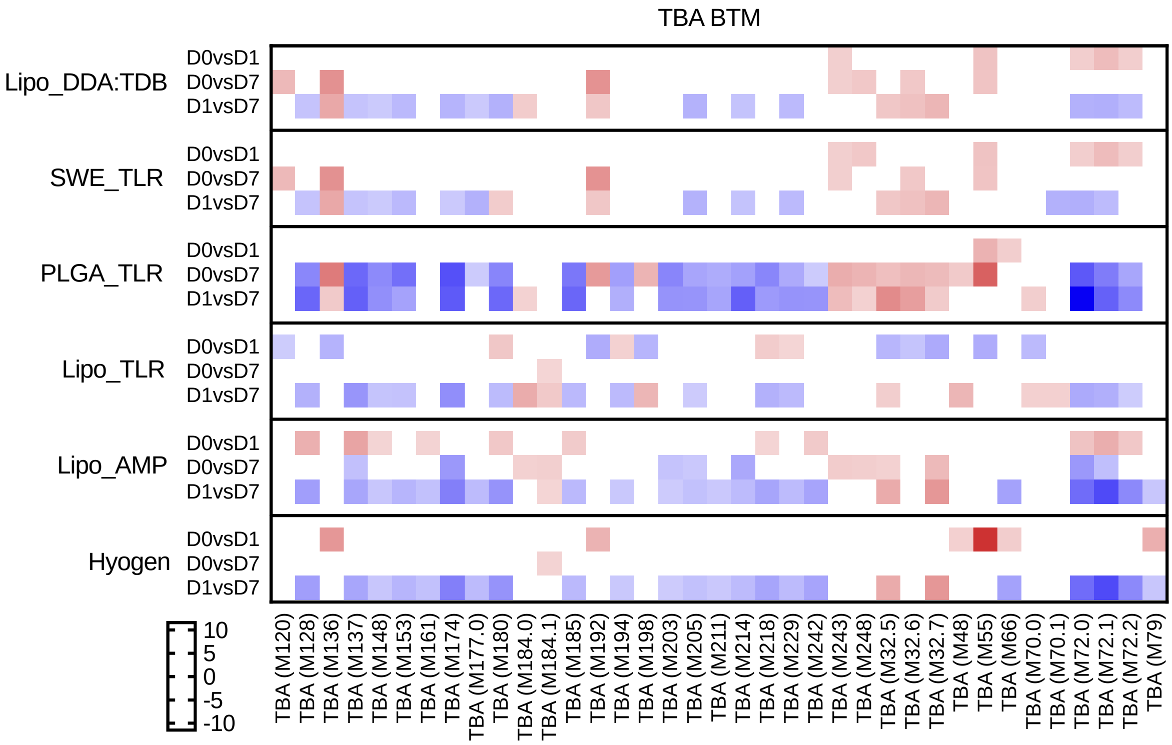

Supplementary Figure 5. TBA BTM induced by vaccines. The heat maps show the vaccine-dependent induction of BTM activity determined for D0 to D1 (D0vsD1), for D0 to D7 (D0vsD7) and for D1 to D7 (D1vsD7) changes in the modules. The values shown were calculated by -log(P-value)*1 for positively enriched BTM and as -log(P-value)*-1 for negatively enriched BTM. A cut-off of an FDR of q < 0.1 was employed. Red colors indicate BTM upregulation and blue downregulation. BTM, blood transcriptional modules.
